# pH-sensitive micelles for the intracellular co-delivery of curcumin and Pluronic L61 unimers for synergistic reversal effect of multidrug resistance

**DOI:** 10.1038/srep42465

**Published:** 2017-02-14

**Authors:** Wei Hong, Hong Shi, Mingxi Qiao, Zehui Zhang, Wenting Yang, Lingying Dong, Fucheng Xie, Chunpeng Zhao, Li Kang

**Affiliations:** 1 Key Laboratory of Zoonosis of Liaoning Province, College of Animal Science and Veterinary Medicine, Shenyang Agricultural University, Dongling Road 120, Shenyang, Liaoning Province, 110866, P.R. China; 2 School of Pharmacy, China Pharmaceutical University, Longmian Avenue 639, Jiangning District, Nanjing, 211198, P.R. China; 3 School of Pharmacy, Shenyang Pharmaceutical University, Wenhua Road 103, Shenyang, Liaoning Province, 110016, P.R. China

## Abstract

Pluronic L61 unimers, which are biomacromolecular modulators, and curcumin, a small-molecule modulator, were co-formulated into pH-sensitive micelles to reveal the full synergistic potential of combination drug treatments to reverse multidrug resistance (MDR). Compared to monotherapy, combined therapy significantly improved the cytotoxicity, cellular uptake and apoptotic effects of doxorubicin (DOX) against MCF-7/ADR cells. In mechanistic studies, both L61 and curcumin enhanced the cytotoxic effect by acting on mitochondrial signalling pathways. The compounds selectively accumulated in the mitochondria and disabled the mitochondria by dissipating the mitochondrial membrane potential, decreasing the ATP levels, and releasing cytochrome *c*, which initiated a cascade of caspase-9 and caspase-3 reactions. Furthermore, both curcumin and L61 down-regulated the expression and function of P-gp in response to drug efflux from the MCF-7/ADR cells. In the MCF-7/ADR tumour-bearing mouse model, intravenous administration of the combined therapy directly targeted the tumour, as revealed by the accumulation of DiR in the tumour site, which led to a significant inhibition of tumour growth without measurable side effects. In conclusion, co-formulation consisting of L61 and curcumin in pH-sensitive micelles induced significant synergistic effects on the reversal of MDR. Therefore, the intracellular co-delivery of various MDR modulators has great potential to reverse MDR in tumours.

Multidrug resistance (MDR) has become a major obstacle to the successful treatment of cancer. Over the last decade, many studies have been performed to elucidate the mechanism of drug resistance and to develop effective therapeutic approaches. MDR is initially attributed to the overexpression of ATP-binding cassette (ABC) transporters, such as P-gp, MRPs, and BCRPs, on the cell membrane, which increase the efflux of drugs from cancer cells^1^,^2^. The development of ABC transporter inhibitors is one of the most widely studied strategies for reversing MDR^3^. However, ABC transporter inhibitors have had only limited success in reversing MDR because MDR is complicated and comprises multiple correlated resistance mechanisms. For example, in addition to the overexpressed ABC transporters, the glutathione/glutathione S-transferase detoxification system is frequently activated in MDR cells. MRP acts in concert with this system, inducing the efflux of glutathione conjugates of xenobiotics from the cells^4^. Another mechanism contributing to MDR involves the sequestration of drugs within cytoplasmic vesicles, followed by the extrusion of the drug out of the cells^5^. However, until now, most previous studies regarding tumour MDR reversal have focused on reversing one resistance mechanism, namely, ABC protein-mediated drug efflux^6^. The complexity of the tumour MDR mechanism accounts for the limited success in reversing tumour MDR to date^7^. Therefore, targeting the delivery of two or more MDR modulators with the ability to reverse multiple pathways is a potentially better strategy for reversing MDR.

Chinese medicines (CMs, including plants, animal parts and minerals) have drawn a great deal of attention in recent years for their potential in the treatment of MDR^8^. Curcumin (diferuloylmethane, CUR) is a naturally occurring polyphenol extracted from turmeric root, *Curcuma longa*. Curcumin is capable of reversing MDR in tumours by regulating the expression and function of ABC transporters^9^,^10^, inhibiting ATPase activity^11^, and modulating NF-κB activity^12^ and specific miRNAs^13^. Recently, mitochondria have been suggested as a promising target by which curcumin induces the apoptosis of MDR cancer cells^14^,^15^. Curcumin can induce the translocation of mitochondrial permeability transition pores (PTPs), resulting in the release of cytochrome *c* from the mitochondria into the cytoplasm, the dissipation of the mitochondrial membrane potential (∆Ψ), the impairment of respiration, the inhibition of ATP synthesis and the activation of the apoptotic protein enzyme caspase-9, which triggers the activation of the downstream caspase-3 protein. These caspases are responsible for cleaving key cellular proteins, which induces apoptosis^16^,^17^. Thus, curcumin could mediate chemo-resistance by sensitizing cancer cells to conventional chemotherapeutic agents^18^. A combination of curcumin with conventional anticancer agents may lead to better treatment outcomes for tumour MDR therapy. Curcumin has been co-administered with various potent chemotherapeutic drugs, such as cisplatin, doxorubicin, paclitaxel, etc., as part of the treatment modalities for many multidrug-resistant cancers^19^,^20^,^21^,^22^.

In addition to curcumin, Pluronics have been identified as the most promising MDR reversal agents due to their effects on reversing several distinct drug resistance mechanisms, including blocking drug efflux transporters^23^,^24^,^25^, changing the microviscosity of cell membranes^26^, reducing the ATP levels in MDR cells^27^, inhibiting the glutathione (GSH)/glutathione (GST) detoxification system^28^, inducing the release of cytochrome *c* and increasing the reactive oxygen species (ROS) levels in the cytoplasm^29^. As shown in mechanistic studies, mitochondria might be potential sites of action for Pluronics. Pluronics can be selectively localized to mitochondria and can reduce the activity of the electron transport chains in mitochondria. The ability of Pluronics to serve as K^+^ ionophores^30^,^31^ and to uncouple oxidative phosphorylation^32^ likely contributed to their anti-metabolic effects. Pluronics may also directly inhibit the NADH dehydrogenase complex by interacting with the hydrophobic sites of this complex in the mitochondrial membrane^33^. The reversal effects are most apparent at copolymer concentrations below the critical micelle concentration (CMC)^34^, suggesting that the Pluronic unimers, i.e., single block copolymer molecules, play a major role in MDR reversal^35^,^36^. In our previous study, the intracellular delivery of Pluronic unimers using pH-sensitive micelles was effective for reversing MDR in tumours^37^,^38^.

Over the last few decades, combination therapy has been adopted in clinics to address the problems associated with single chemotherapeutic MDR cancer treatments. Combination therapy generally refers to two or more therapeutic agents or MDR modulators that are simultaneously co-delivered. By combining two or more agents, the side effects associated with large doses of single drugs can be overcome by synergistically countering different biological signalling pathways, allowing patients to be treated with a low dose of each compound or for researchers to assess context-specific multi-target mechanisms. In addition, the co-delivered drugs target the same cellular pathways that may function synergistically to achieve greater therapeutic efficacy and better selectively^39^. Considering the complexity of MDR, the combination of two MDR modulators could hold great promise for synergistically reversing MDR^40^,^41^,^42^.

In the present study, an endosomal, pH-sensitive, mixed micellar delivery system with a folate targeting ligand based on the pH-sensitive copolymer PHis-PLA-PEG-PLA-PHis and Pluronic F127 (F-pHSM-L61/CUR/DOX) was constructed for the intracellular co-delivery of the small modulating molecule curcumin and the macromolecular modulating molecules Pluronic unimers. A small proportional Pluronic F127 was conjugated with folate to actively target the mixed micelles. The relatively long hydrophilic polyethylene oxide (PEO) block (4500 Da) of Pluronic F127 ensured prolonged circulation of the micelles and manipulated the triggering pH (pH 5.5). The pH-sensitive copolymer PHis-PLA-PEG-PLA-PHis was responsible for disrupting the micellar structure in early or late endosomes, triggering the release and trafficking of the Pluronic L61 unimers and curcumin to the cytosol via copolymer-facilitated endosomal escape to exert their synergistic MDR reversal effect (Fig. 1). Here, we reported pH-sensitive micelles that are scalable, simultaneously carry Pluronic L61 unimers and curcumin, and exhibit increased cytotoxicity, cellular uptake and cell apoptosis. The systemic administration of the pH-sensitive micelles significantly inhibited tumour growth and limited systemic toxicity. Mechanistically, the Pluronic L61 unimers and curcumin co-formulated pH-sensitive micelles exhibited a synergistic MDR reversal effect by inhibiting mitochondrial signalling pathways and the expression and function of P-gp. Thus, even a conventional anti-cancer drug, doxorubicin, could still effectively treat multidrug-resistant cancer following the intracellular co-delivery of two MDR reversal agents, due to a synergistic MDR reversal effect.

## Materials and Methods

### Reagents

Doxorubicin (DOX) was purchased from Beijing HuaFeng United Technology Co., Ltd. (Beijing, China). Curcumin (CUR), polyethylene glycol (PEG) (*M*_*n*_: 2000 g/mole), N,N′-Carbonyldiimidazole (CDI), 3-(4,5-dimethyl-thiazol-2-yl)-2,5-diphenyl-tetrazolium bromide (MTT), rhodamine B isothiocyanate (RITC) and Hoechst 33258 dye were purchased from Sigma (St. Louis, MO, USA). RPMI 1640 medium without folic acid and foetal bovine serum (FBS) were purchased from Gibco BRL (Gaithersburg, MD, USA). The rabbit polyclonal P-glycoprotein 1 antibody, mouse monoclonal β-actin antibody, mouse polyclonal cytochrome *c* antibody, cleaved poly ADP ribose polymerase (PARP), horseradish peroxidase-conjugated goat anti-rabbit IgG and horseradish peroxidase-conjugated goat anti-mouse IgG were purchased from Boster Biological Technology, Ltd. (Wuhan, China). Cytoplasmic and mitochondrial protein extraction kits were purchased from Sangon Biotech (Shanghai, China). The mouse monoclonal anti-cytochrome *c* oxidase subunit IV (COX IV) antibody was purchased from AmyJet Scientific Inc. (Wuhan, China). An Annexin V-FITC (fluorescein isothiocyanate)/propidium iodide (PI) apoptosis detection kit, a caspase-3 activity assay kit, a caspase-9 activity assay kit, a cell mitochondria isolation kit, an ATP assay kit and a mitochondrial membrane potential assay kit with JC-1 were purchased from Beyotime^®^ Biotechnology Co., Ltd. (Nantong, China). Pluronic F127 and Pluronic L61 were kindly supplied by BASF Ltd. (Shanghai, China). All other reagents and chemicals were of analytical or chromatographic grade and were purchased from Concord Technology (Tianjin, China).

### Cells

The MCF-7/ADR multidrug-resistant human breast cancer cell line was purchased from Jiangsu KeyGEN Biotech Corp., Ltd. (Nanjing, Jiangsu, China). MCF-7/ADR cells were cultured in RPMI 1640 medium with 10% FBS, 100 IU/mL penicillin, 100 μg/mL streptomycin sulphate and 1000 ng/mL doxorubicin. The cells were cultured in a CO_2_ incubator with 5% CO_2_ at 37 °C. All experiments were performed while the cells were in the logarithmic phase of growth.

### Animals

Female BALB/c nude mice (20 ± 2 g) supplied by the Department of Experimental Animals at Shenyang Pharmaceutical University (Shenyang, Liaoning, China) were acclimated at 25 °C and 55% humidity under natural light/dark conditions. The mice were fed a diet lacking folic acid for 2 weeks prior to the study and for the duration of the study. All animal experiments were conducted in accordance with the guidelines evaluated and approved by the ethics committee of Shenyang Pharmaceutical University.

### Tested formulations

The following formulations were tested: DOX solution; CUR solution; Pluronic L61 solution; Pluronic L61 +CUR solution; F-pHSM-L61/CUR/DOX: folate-mediated endosomal pH-sensitive mixed micelles composed of PHis-PLA-PEG-PLA-PHis, folate-Pluronic F127 and Pluronic F127 loaded with DOX, curcumin and Pluronic L61 unimers; F-pHSM-L61/DOX: folate-mediated endosomal pH-sensitive mixed micelles composed of PHis-PLA-PEG-PLA-PHis, folate-Pluronic F127 and Pluronic F127 loaded with DOX and Pluronic L61 unimers; F-pHSM/CUR/DOX: folate-mediated endosomal pH-sensitive mixed micelles composed of PHis-PLA-PEG-PLA-PHis, folate-Pluronic F127 and Pluronic F127 loaded with DOX and curcumin; and F-pHSM/DOX: folate-mediated endosomal pH-sensitive mixed micelles composed of PHis-PLA-PEG-PLA-PHis, folate-Pluronic F127 and Pluronic F127 loaded with DOX.

### Synthesis of PHis-PLA-PEG-PLA-PHis, folate-Pluronic F127 and RITC-Pluronic L61 copolymers

The copolymers used in this work were all homemade, as previously reported^37^,^38^.

### Preparation and characterization of DOX-loaded micelles

F-pHSM-L61/CUR/DOX was prepared using a thin-film hydration method. First, doxorubicin (DOX) hydrochloride (30 mg) was stirred with triethylamine (molar ratio, 1/3) in acetonitrile (20 mL) overnight to obtain the DOX base. Then, the DOX base (20 mg) and CUR (40 μg) were blended with 380 mg of copolymer mixtures in 20 mL of acetonitrile. The mixture was sonicated for 30 minutes to allow dissolution. The solvent was evaporated in a rotary evaporator at 40 °C to obtain a thin film. The residual acetonitrile in the film was further removed under vacuum overnight at room temperature. The resulting thin film was hydrated with 10 mL of PBS (pH 8.0) for 30 min to obtain a micellar solution. The micellar solution was filtered through a 0.22-μm film to remove the unincorporated DOX and CUR aggregates. Other tested micelles (F-pHSM/CUR/DOX, F-pHSM-L61/DOX and F-pHSM/DOX) were fabricated with the corresponding copolymer mixtures and drugs using the same procedure.

The drug loading coefficient (DL%) and entrapment efficiency (EE%) were measured using a multifunctional microplate reader (Tecan, Austria) and calculated using Eqs 1 and 2, respectively. The λex (442 nm)/λem (475 nm) ratio was used to detect CUR, and the λex (488 nm)/λem (575 nm) ratio was used to detect DOX.


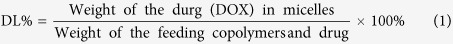



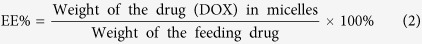


The particle size distributions and Zeta potentials of the prepared micelles were measured with dynamic light scattering (DLS, Zetasizer Naso ZS, Malvern, UK) at 25 °C after equilibration for 5 min. Each freshly prepared sample was placed into a quartz cuvette without additional treatment. The size of each sample was measured in triplicate.

The morphology of F-pHSM-L61/CUR/DOX was investigated by transmission electron microscopy (JEM-1230, Japan) operating at an acceleration voltage of 80 kV. TEM samples were prepared by dipping a copper grid into the micelle solution, followed by staining with a phosphotungstic acid solution (2%, w/v) for approximately 15 s. Subsequently, the sample was allowed to slowly dry in air at room temperature for 2 h before the TEM observation.

### *In vitro* drug release from the copolymer micelles

The release behaviours of CUR and DOX from the F-pHSM-L61/CUR/DOX, F-pHSM/CUR/DOX, F-pHSM/CUR and F-pHSM/DOX formulations were investigated using a dialysis method with 80 mL of phosphate buffer (pH 7.4 and pH 5.0) at 37 °C under sink conditions (0.5 wt% Tween 80). At predetermined time intervals, 0.2 mL aliquots were withdrawn and replaced with an equal volume of fresh medium. The CUR and DOX concentrations were quantified using fluorescence spectrophotometry and a multifunctional microplate reader (Tecan, Austria). The λex (442 nm)/λem (475 nm) ratio was used to detect CUR, and the λex (488 nm)/λem (575 nm) ratio was used to detect DOX. Each release experiment was performed in triplicate, and the cumulative DOX and CUR release were plotted as a function of time.

### *In vitro* cell cytotoxicity

The *in vitro* cytotoxicity of the drug-loaded micelles towards MCF-7/ADR cells was evaluated using the MTT method^43^. The cells were seeded in 96-well plates at a density of 5 × 10^3^ cells per well and incubated for 24 h. Then, the growth medium was replaced with fresh medium containing the indicated concentration of the tested formulations (F-pHSM-L61/CUR/DOX, F-pHSM/CUR/DOX, F-pHSM-L61/DOX, F-pHSM/DOX and DOX solution). Control wells were treated with an equivalent volume of DOX-free medium. The cells were incubated at 37 °C for 48 h. After incubation, the wells were rinsed with PBS, the MTT solution (5 mg/mL) was added to each well, and the plate was incubated for 4 h. Finally, the medium was completely removed, and 150 μL of dimethyl sulphoxide (DMSO) was added to each well to dissolve the purple formazan crystals. The absorbance was measured at 570 nm using a multifunctional microplate reader (Tecan, Austria). The IC_50_ values were calculated using a nonlinear regression analysis, and the MDR reversal effect was assessed by quantifying the IC_50_ values of the tested formulations.

### Confocal laser scanning microscopy (CLSM)

MCF-7/ADR cells were seeded on a cover-slide system at a density of 2.5 × 10^4^ cells/well and placed in a humidified incubator for 24 h. The tested formulations (F-pHSM-L61/CUR/DOX, F-pHSM/CUR/DOX, F-pHSM-L61/DOX, F-pHSM/DOX and DOX solution) were then added, and the cells were further incubated for 0.5 h, 1 h, 2 h, 4 h, 8 h and 12 h. Thereafter, the cells were washed three times with cold PBS and stained with 10 μM Hoechst 33258 for 10 min to visualize the nuclei. Then, the cells were fixed with 4% paraformaldehyde for 30 min. Cells treated with regular medium were used as a negative control. Images were captured using a confocal laser scanning microscope (CLSM, Olympus FV1000-IX81, Japan). An excitation wavelength of 560 nm and an emission wavelength of 600 nm were used to detect DOX, an excitation wavelength of 488 nm and an emission wavelength of 550 nm were used to detect CUR, and an excitation wavelength of 405 nm and an emission wavelength of 500 nm were used to detect Hoechst 33258.

### Apoptosis assay

Apoptosis assay was detected using an Annexin V-FITC/PI apoptosis detection kit. After attaining 90% confluency, the cells were treated with the tested formulations as previously described. After being exposed to the tested formulations for 24 h, cells were harvested by trypsinization, washed with ice-cold PBS three times, and then immediately centrifuged. The supernatant was discarded, and the cells were re-suspended in PBS (5 × 10^5^–1 × 10^6^ cells/mL). The cell solution (1 mL) was centrifuged for 5 min at 4 °C and then re-suspended in a mixture of 195 μL of Annexin V-FITC binding buffer, 5 μL of Annexin V-FITC solution and 10 μL of PI solution. The samples were mixed gently and incubated at room temperature for 15 min in the dark. After the incubation, the samples were immediately analysed using a BD FACSCalibur flow cytometer (FACSCAN, Becton Dickinson, San Jose, CA, USA). Cells cultured in media containing F-pHSM-L61/CUR/DOX without staining were also analysed in the same way as the auto-fluorescence reference. Triplicate samples were analysed for each experiment.

### Drug content in the mitochondria

The drug content in the mitochondrial fraction was quantified using a BD FACSCalibur flow cytometer (FACSCAN, Becton Dickinson, San Jose, CA, USA). MCF-7/ADR cells were cultured and then treated with the tested formulations (curcumin solution, RITC-Pluronic L61 unimer solution, mixed RITC-Pluronic L61 unimers/CUR solution, and F-pHSM-L61/CUR) for 2 h with 5% CO_2_ at 37 °C. The cells were harvested and washed twice with cold PBS (pH 7.4). The mitochondria were isolated with the Cell Mitochondria Isolation Kit, according to the manufacturer’s instructions. The amounts of the formulations taken up by the mitochondria were measured using a FACSCAN flow cytometer; approximately 1 × 10^4^ events were collected and presented as the fluorescence intensity. An excitation wavelength of 425 nm and an emission wavelength of 530 nm were used to detect CUR, and an excitation wavelength of 558 nm and an emission wavelength of 586 nm were used to detect RITC-Pluronic L61 unimers. Each assay was performed in triplicate.

### ATP content and mitochondrial membrane potential assays

Confluent MCF-7/ADR cells were treated with the tested formulations (mixed Pluronic L61 unimers/CUR solution, Pluronic L61 unimer solution, CUR solution, F-pHSM-L61/CUR, F-pHSM/CUR, F-pHSM-L61 and F-pHSM) for 2 h to determine the intracellular ATP content. Then, the cells were washed twice with ice-cold PBS and solubilized into cell lysates; they were then immediately centrifuged (12000 × *g*) at 4 °C for 10 min. The supernatant was collected to quantify the ATP concentrations using a luciferin/luciferase assay kit. Light emission was measured with an Ultra-Weak Luminescence Analyser (model BPCL, China). The raw data were converted to ATP concentrations using the standard calibration curve. The ATP content was normalized to the protein content in each sample, as determined using a BCA kit. Blank medium was used as a control.

Changes in the mitochondrial membrane potential were assessed using the lipophilic cationic membrane potential-sensitive dye JC-1 (5,5′,6,6′–tetrachloro-1,1′,3,3′- tetraethyllenzimidazolycarbocyanine iodide)^44^. Briefly, confluent MCF-7/ADR cells were treated with the different formulations (mixed Pluronic L61 unimers/CUR solution, Pluronic L61 unimer solution, CUR solution, F-pHSM-L61/CUR, F-pHSM/CUR, F-pHSM-L61 and F-pHSM) for 2 h and then washed three times with cold PBS. The trypsinized cells (5 × 10^5^) were suspended in 500 μL of diluted JC-1 staining solution for 20 min. Then, the cells were rinsed twice with physiological saline. Subsequently, the cells were suspended in 500 μL of JC-1 staining buffer and immediately measured using a multifunctional microplate reader (Tecan, Austria) at λ_ex_ (488 nm)/λ_em_ (590 nm) for red fluorescence or λ_ex_ (488 nm)/λ_em_ (530 nm) for green fluorescence, according to the manufacturer’s instructions. CUR solution was also analysed as the auto-fluorescence reference. The obtained values were then expressed as the average ratio of the JC-1 red/green (R/G) signal intensities (n = 6).

### Caspase activation

Caspase-3 and caspase-9 activities in the MCF-7/ADR cells were determined using peptide substrates that emit fluorescence when they are cleaved by a specific protease^45^. Briefly, MCF-7/ADR cells were cultured for 24 h. Then, the cells were treated with the different formulations (mixed Pluronic L61 unimers/CUR solution, Pluronic L61 unimer solution, CUR solution, F-pHSM-L61/CUR, F-pHSM/CUR, F-pHSM-L61 and F-pHSM). Control experiments were performed by adding blank medium. After a 24-h incubation, the cells were harvested and lysed. The cell lysates were centrifuged at 10000 rpm for 5 min at 4 °C. The supernatants were stored and treated with caspase-3 and caspase-9 substrates. Caspase-3 and caspase-9 activities were measured at 405 nm on a microplate reader, and the activity ratio was calculated according to the manufacturer’s instructions. Each assay was performed in triplicate.

### Accumulation and efflux of Rh123

Rh 123 accumulation was measured as previously described^46^. Briefly, MCF-7/ADR cancer cells were seeded in 6-well plates at a density of 5 × 10^3^ cells per well and incubated for 24 h. Then, the growth medium was replaced with fresh medium containing the indicated concentration of a formulation (F-pHSM-L61/CUR, F-pHSM/CUR, F-pHSM-L61 and F-pHSM) and 32 μM Rh 123. Following Rh 123 accumulation for 1 h, the cells were washed three times with PBS and solubilized in 1% Triton X-100. Aliquots were removed to analyse the cellular dye (Rh 123) content using a multifunctional microplate reader (Tecan, Austria) at λex = 505 nm and λem = 540 nm. CUR solution was also analysed as the auto-fluorescence reference. The cellular protein content was determined using a BCA kit. Fluorescence intensities were normalized to the protein content in each well. P-gp activity was expressed as a percentage of dye uptake in the formulation-treated vs. untreated cells.

The cells were first treated with 32 μM Rh 123 for 1 h, and then, the medium was replaced with fresh medium containing the F-pHSM-L61/CUR, F-pHSM/CUR, F-pHSM-L61 or F-pHSM formulation to examine the Rh 123 efflux. Following efflux intervals of 1 h, the medium was removed, and the cells were washed three times with PBS and prepared for testing, as described above.

### Western blot analysis

The cytochrome *c* protein content in the cytoplasmic and mitochondrial fractions and the P-gp expression were determined using Western blot analyses. Briefly, after a 24 h incubation, MCF-7/ADR cells were treated with the different tested formulations (mixed Pluronic L61 unimers/CUR solution, Pluronic L61 unimer solution, CUR solution, F-pHSM-L61/CUR, F-pHSM/CUR, F-pHSM-L61 and F-pHSM) for 24 h. Control experiments were performed by adding blank medium. Then, the cells were harvested and treated with lysis buffer. Cytoplasmic and mitochondrial proteins were extracted separately using a cytoplasmic and mitochondrial protein extraction kit (Sangon, Shanghai, China). Membrane proteins were extracted using a membrane protein extraction kit (Beyotime, China). The cytoplasmic, mitochondrial and membrane proteins were quantified using a BCA protein assay kit (Beyotime, China). Protein samples (100 μg) were separated using SDS-PAGE and then electrophoretically transferred to a nitrocellulose membrane. The nitrocellulose membrane was blocked with skim milk for 2 h at room temperature and incubated with specific primary antibodies over night at 4 °C, followed by an incubation with secondary antibodies for 1 h at room temperature, respectively. Specific protein bands were visualized with enhanced chemiluminescence detection reagents and a gel imaging system (Tanon 5200, Tanon Science & Technology Co., Ltd., Shanghai, China). Band intensities were measured, and the protein signals were normalized to the β-actin or COX IV levels.

### DiR fluorescence real-time tumour imaging

MCF-7/ADR cells were transplanted into female BALB/c nude mice by subcutaneously injecting 100 μL of 1 × 10^7^ cells suspended in cell culture media. When the tumour was approximately 150–200 mm^3^, 0.2 mL of the DiR-loaded F-pHSM-L61/CUR, F-pHSM/CUR, F-pHSM-L61 or F-pHSM formulation was intravenously injected through the tail vein. The time-dependent biodistribution in the MCF-7/ADR tumour-bearing nude mice was imaged at 1 h, 2 h, 4 h, 6 h, 8 h, 12 h, 24 h, 36 h, 48 h, 60 h and 72 h post-injection using the Kodak *In Vivo* Imaging System FX PRO (Carestream Health, Inc., USA). The mice that had been anesthetized via the inhalation of Gerolan Sol were automatically placed in the imaging chamber for scanning. The tumour-bearing mice were euthanized after 72 h, followed by the immediate removal of the tumour masses and the heart, liver, spleen, lungs, kidneys and brain to further observe the distribution of the tumour masses in the major organs. The fluorescence intensities in different tissues were photographed. An excitation wavelength of 748 nm and emission wavelength of 780 nm were used to detect DiR.

### Tumour growth inhibition assay

MCF-7/ADR cells were transplanted into female BALB/c mice as described above. The mice were randomly divided into six groups (n = 6) when the tumours reached approximately 100 mm^3^ in volume. Then, the mice were intravenously injected with a) saline, b) a DOX solution, c) F-pHSM-L61/CUR, d) F-pHSM/CUR, e) F-pHSM-L61, f)F-pHSM-L61/CUR/DOX, g) F-pHSM/CUR/DOX, h) F-pHSM-L61/DOX, and i) F-pHSM/DOX through the tail vein at a dose of 10 mg/kg DOX every 2 days for 29 days. The tumour sizes and the body weights of the mice were measured every 2 days. At the end of the experiment, all mice were euthanized, and the tumours were harvested and weighed. The anti-tumour activity was assessed by the tumour volume (V), which was calculated using the following equation: V (mm^3^) = (LW^2^)/2, where length (L) was the longest diameter and width (W) was the shortest diameter perpendicular to the length. Tumour volumes were recorded on day 29, and tumour growth inhibition (TGI) was calculated using Eq. 3:


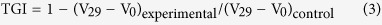


where V_0_ = the volume of the tumour on day 0, and V_29_ = the volume of the tumour on day 29.

The blood samples were obtained at the end of the experiment and analyzed by a Hitachi 7100 Automatic biochemical analyzer (Japan). Tumor tissues were excised, minced, and homogenized in protein lysate buffer. Debris was removed by centrifugation. The P-gp level and cleaved PARP in nanoparticle treated xenograft BALB/c mice were also determined using Western blot analyses as described above.

### Statistical analysis

All experiments were performed at least three times. Quantitative data are presented as the mean ± standard deviations (S.D). Statistical comparisons among ≥3 groups were performed using an analysis of variance (ANOVA) and comparisons between 2 groups were performed using Student’s t-test. *P*-values < 0.05 and <0.01 were considered statistically significant.

## Results and Discussion

### Composition and characterization of the pH-sensitive micelles

For the intracellular co-delivery of Pluronic L61 unimers and curcumin in MDR cells, endosomal pH (pH ~5.5)-sensitive micelles conjugated with folate (F-pHSM) were constructed using a copolymer composed of PHis-PLA-PEG-PLA-PHis and Pluronic F127. The weight ratio of PHis-PLA-PEG-PLA-PHis/Pluronic F127 was set to 80/20 to provide the desired endosomal triggering pH (~5.5), as shown in our previous study^37^. The optimal curcumin loading content in the tested micelles was set to 1 × 10^−2^ wt% using the MTT method, and the details are provided in the Supplementary Information. The effects of the Pluronic L61 unimer content loaded into the F-pHSM-L61 formulation on cytotoxicity and cellular uptake were evaluated using the MTT method and flow cytometry, respectively. The optimum loading content of Pluronic L61 unimers was 3 × 10^−4^ wt%, and the details are provided in the Supplementary Information.

**Figure 2:**
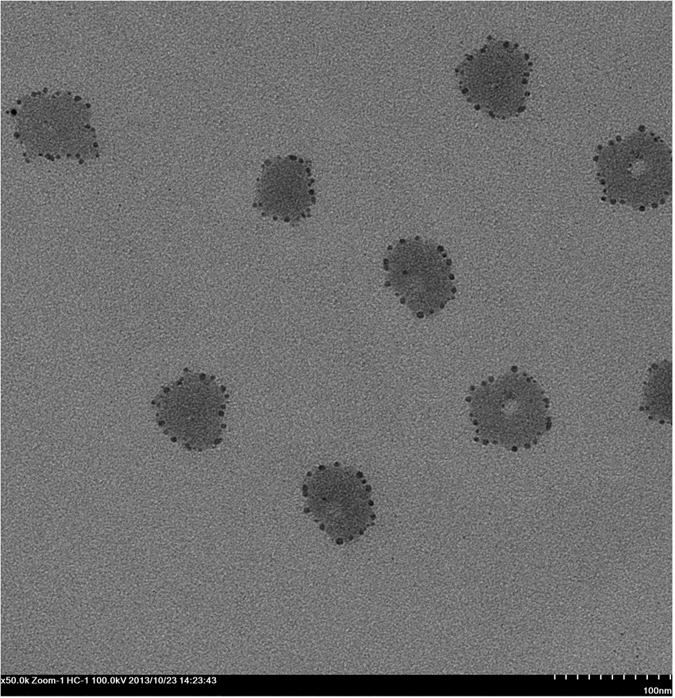
TEM images of F-pHSM-L61/CUR/DOX.

The particle size, the size distribution, the surface charge, the drug loading coefficient (DL%) and the entrapment efficiency (EE%) were determined for each formation. The corresponding physical characterizations are summarized in Table 1. The hydrodynamic diameters and the surface charges (Zeta potentials) of the micellar formulations containing only curcumin, only doxorubicin and both curcumin and doxorubicin measured using the dynamic light scattering technique ranged between 180–230 nm and −5~−6 mV, respectively. Small micelles (<200 nm) and negative surface charges are beneficial for prolonging *in vivo* circulation and the subsequent passive or active targeting^47^. The morphology of the F-pHSM-L61/CUR/DOX formulation was spherical with a smooth surface and a uniform size distribution (Fig. 2). The amount of drug loaded and the entrapment efficiency of DOX in the F-pHSM-L61/CUR/DOX formulation were 4.27% and 85.4%, respectively. When CUR was incorporated into the polymerized formulation, the entrapment efficiency of DOX in the micelles decreased from 90.3% to 85.4%. Therefore, the incorporation of CUR into the polymerization medium during the preparation of the F-pHSM-L61/CUR/DOX formulation hindered the encapsulation of doxorubicin into the micelles, which was in good agreement with a previous report^48^. The incorporation of Pluronic L61 into the polymerization medium did not significantly affect the DOX entrapment efficiency in the micelles; the DOX (EE%) only decreased from 90.7% to 90.3%.

**Figure 3:**
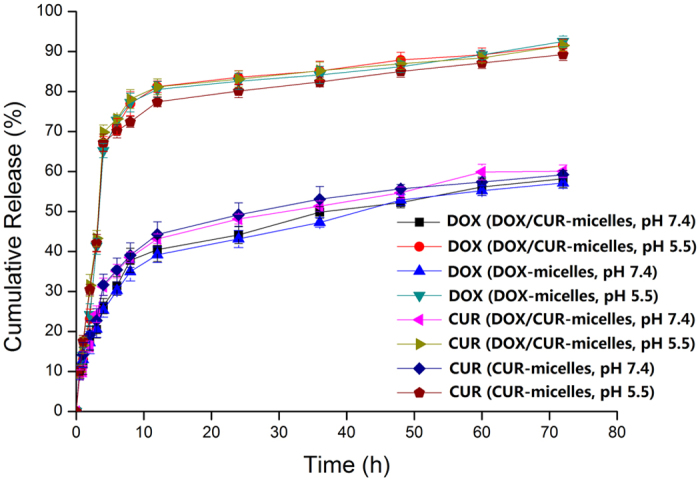
Cumulative release of DOX and CUR from the pH-sensitive micelles at various pH values (pH 7.4 and 5.5) at 37 °C.

An *in vitro* dissolution test was performed to verify the pH-triggered drug release profiles. Prior to conducting these release assays, DOX and CUR were verified to freely diffuse through the dialysis membrane and the sink conditions were respected. As shown in Fig. 3, the *in vitro* profiles of DOX and CUR release from the DOX/CUR/L61-micelles showed biphasic release at pH 7.4, with rapid release in the first 4 h, followed by sustained release until 48 h. A large increase in the drug release rate occurred when the pH was reduced to pH 5.5. The sustained release profile at neutral pH (pH 7.4) ensures that the copolymer micelles deliver most of the two drugs to the tumour site. At acidic pH, the imidazole groups of the PHis block begin to protonate, inducing micellar structure destabilization and accelerated drug release.

### Intracellular co-delivery of Pluronic L61 and curcumin using pH-sensitive micelles

**Figure 4:**
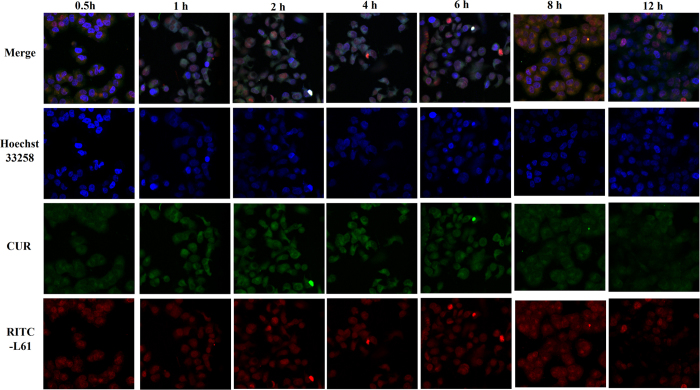
Confocal images of the intracellular localizations of RITC-Pluronic L61 unimers and curcumin in MCF-7/ADR cells. The cells were incubated with F-pHSM-L61/CUR for 0.5 h, 1 h, 2 h, 4 h, 6 h, 8 h and 12 h at 37 °C.

The subcellular distributions of Pluronic L61 unimers and curcumin were studied by CLSM using triple labelling with a nucleus-selective dye (Hoechst 33258, blue), fluorescent Pluronic L61 unimers (RITC- Pluronic L61, red) and curcumin (green) to investigate the intracellular co-delivery of Pluronic L61 and curcumin. The autofluorescence of the MCF-7/ADR cancer cells was negligible under the conditions used to detect DOX and CUR. Images of the fluorescently stained MCF-7/ADR cells that had been incubated with the blank F-pHSM-L61/CUR formulation for different time intervals are presented in Fig. 4. The overlapping green (curcumin) and red (Pluronic L61 unimers) fluorescence in the MCF-7/ADR cells at 0.5 h of incubation indicates the rapid internalization of the micelles and the efficient co-delivery of the Pluronic L61 unimers and curcumin into the MDR cells. In addition, the fluorescence intensities of the RITC-Pluronic L61 unimers and curcumin in the cytosol of the MCF-7/ADR cells increased with the time of incubation (Fig. 4). Thus, the Pluronic L61 unimers and CUR were efficiently delivered to the cytosol by the pH-sensitive micelles, which was attributed to folate-mediated endocytosis into endosomes, endosomal pH-triggered drug release and copolymer-facilitated endo/lysosomal escape of the payloads.

### *In vitro* cytotoxicity, cellular uptake and cell apoptosis induced by the pH-sensitive micelles

**Figure 5:**
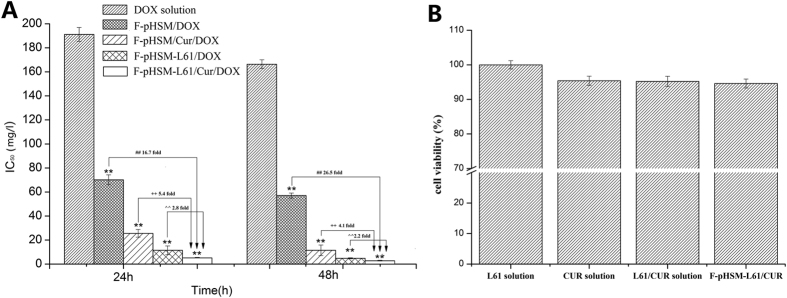
*In vitro* cytotoxicity of the DOX solution and the F-pHSM/DOX, F-pHSM/CUR/DOX, F-pHSM-L61/DOX and F-pHSM-L61/CUR/DOX formulations towards MCF-7/ADR cells after 24 h and 48 h incubations (mean ± S.D., n = 6). **P < 0.01: significantly different from the DOX solution, ^##^*P* < 0.01: significantly different from the F-pHSM/DOX-treated cells, ^++^*P* < 0.01: significantly different from the F-pHSM/CUR/DOX-treated cells, **^^***P* < 0.01: significantly different from the F-pHSM-L61/DOX-treated cells (**A**). The cell viabilion (3 × 10^−4^ wt%), CUR solution (1 × 10^−2^ wt%), CUR(1 × 10^−2^ wt%) + L61(3 × 10^−4^ wt%) solution and F-pHSM-L61/CUR (L61: 3 × 10^−4^ wt%, CUR: 1 × 10^−2^ wt%) (**B**).

The MTT assay was first performed to evaluate the cytotoxicity of the various micellar formulations (F-pHSM-L61/CUR/DOX, F-pHSM-L61/DOX, F-pHSM/CUR/DOX and F-pHSM/DOX) towards the MCF-7/ADR cells. As shown in Fig. 5A, the growth of MCF-7/ADR cells was significantly inhibited when the cells were incubated with the F-pHSM-L61/CUR/DOX formulation. The F-pHSM-L61/CUR/DOX formulation showed a clear dose-dependent cytotoxicity and the lowest IC_50_ among the micellar formulations. The IC_50_ of the F-pHSM-L61/CUR/DOX formulation was 2 times less than the F-pHSM-L61/DOX formulation and 4 times less than the F-pHSM/CUR/DOX formulation. Moreover, the *in vitro* cytotoxicities of the CUR solution, the Pluronic L61 unimer solution, the CUR+ L61 solution and the blank micelles (F-pHSM-L61/CUR) were also evaluated. All of the tested groups showed no visible cytotoxicity towards the MCF-7/ADR cells after 48 h of incubation with the indicated concentrations (cell viability >95%, Fig. 5B), suggesting that the co-delivery of CUR and Pluronic L61 unimers with DOX could enhance the cytotoxic activity of DOX through a “synergistic” MDR reversal effect.

**Figure 6:**
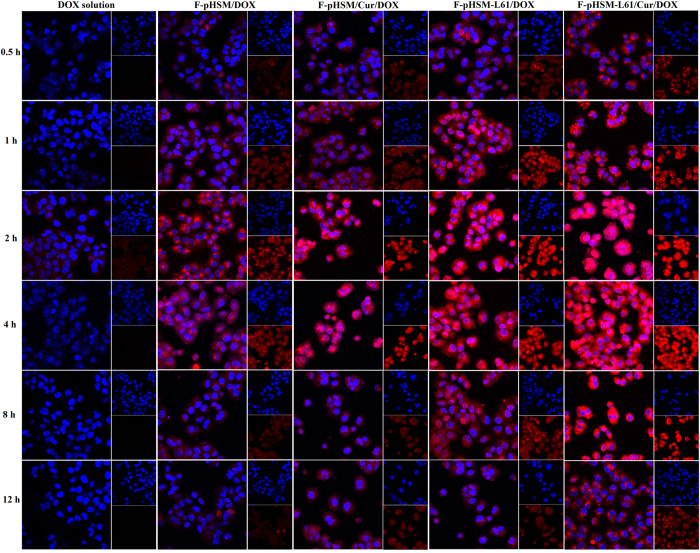
Confocal images of MCF-7/ADR cells that had been incubated with the DOX solution or the F-pHSM/DOX, F-pHSM/CUR/DOX, F-pHSM-L61/DOX or F-pHSM-L61/CUR/DOX formulations for 0.5 h, 1 h, 2 h, 4 h, 8 h and 12 h at 37 °C.

The efficiency of uptake of the different micellar formulations (F-pHSM-L61/CUR/DOX, F-pHSM-L61/DOX, F-pHSM/CUR/DOX and F-pHSM/DOX) by the MCF-7/ADR cells was evaluated using CLSM. As shown in Fig. 6, F-pHSM-L61/CUR/DOX showed time-dependent DOX accumulation and a broad distribution of DOX in the MCF-7/ADR cells during the incubation. The DOX fluorescence intensity increased from 0.5 h to 1 h and reached the highest level at 2 h. The relatively strong fluorescence intensity lasted for more than 12 h. The red fluorescence of DOX stained the entire MCF-7/ADR cell, including the cell nucleus, indicating that the F-pHSM-L61/CUR/DOX formulation was abundantly taken up by the cells and that DOX was effectively released into the cytoplasm. Clearly, the folate-targeting motif on the surface of the F-pHSM-L61/CUR/DOX formulation facilitated the cellular uptake of micelles via specific receptor-mediated endocytosis. Afterward, the pH-sensitive block PHis induced disassembly of the micelles and facilitated endo/lysosomal escape, leading to the release of the payloads (DOX, curcumin and Pluronic L61 unimers) into the cytoplasm. Finally, due to the synergistic MDR reversal effect of curcumin and the Pluronic L61 unimers, the released DOX specifically diffused and accumulated in the nuclei of the cancer cells, inducing apoptosis and cell death. For the control, although the F-pHSM-L61/DOX and F-pHSM/CUR/DOX formulations revealed similar DOX distributions in the cytosol and nuclear compartments compared with the F-pHSM-L61/CUR/DOX formulation, the fluorescence intensity was markedly weaker than the F-pHSM-L61/CUR/DOX formulation. The F-pHSM/DOX formulation displayed the weakest fluorescence intensity among all of the micelles, due to the absence of MDR-modulating agents.

**Figure 7:**
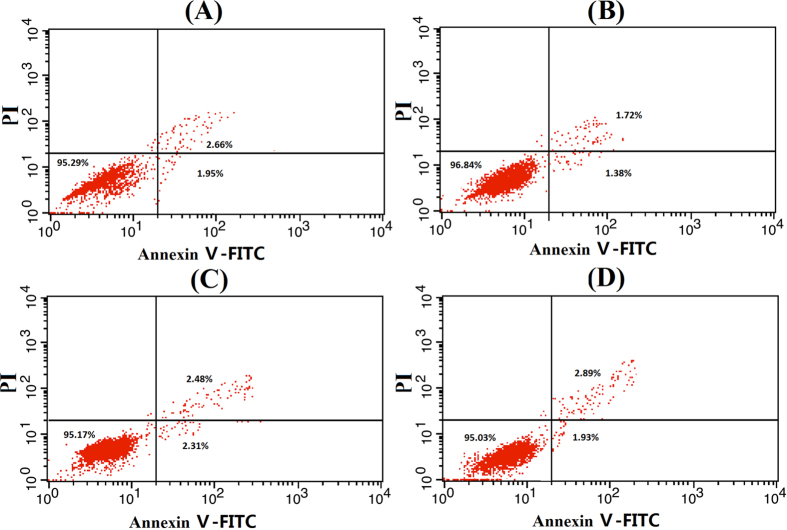
Apoptosis of MCF-7/ADR cells treated with. Curcumin solution (**A**), Pluronic L61 unimers solution (**B**), Cur+ L61 solution (**C**) and F-pHSM-L61/CUR (**D**) at the used concentrations (CUR: 1 × 10^−2^ wt% and L61: 3 × 10^−4^ wt%).

**Figure 8:**
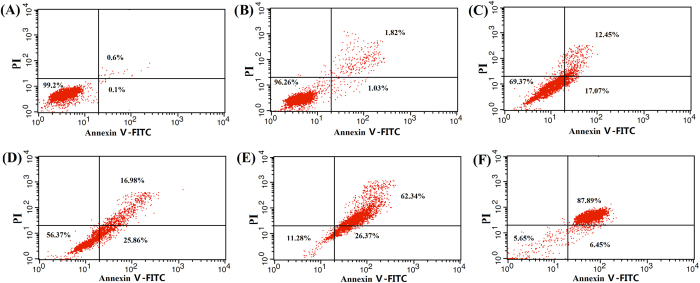
Synergistic effect of micelle-encapsulated Pluronic L61 unimers and curcumin on apoptosis of MCF-7/ADR cells treated with saline (**A**), F-pHSM (**B**), F-pHSM/DOX (C), F-pHSM/CUR/DOX (**D**), F-pHSM-L61/DOX (**E**) and F-pHSM-L61/CUR/DOX (**F**).

Annexin V and PI were used to detect cell apoptosis and necrosis. Four distinct phenotypes were distinguished: viable (lower left quadrant, Q3), early apoptotic (lower right quadrant, Q4), late apoptotic and necrotic (upper right quadrant, Q2) and damaged cells (upper left quadrant, Q1). As shown in Fig. 7, the CUR solution, Pluronic L61 unimers solution, CUR+ L61 solution and F-pHSM-L61/CUR did not cause obvious apoptosis and necrosis of MCF-7/ADR cancer cells at the used concentrations (CUR: 1 × 10^−2^ wt% and L61: 3 × 10^−4^ wt%), which was in good correlation with the *in vitro* cytotoxic assay. The induction of apoptosis after treatment with the different micelle formulations was detected and quantified by flow cytometry (Fig. 8). Of the cells treated with the F-pHSM-L61/CUR/DOX formulation, 87.89% and 6.45% corresponded to late apoptotic and necrotic (Annexin V-FITC^+^PI^+^) and early apoptotic (Annexin V-FITC^+^PI^−^) populations, respectively, compared to 62.34% and 26.37% of the cells treated with the F-pHSM-L61/DOX formulation, 16.98% and 25.86% of the cells treated with F-pHSM/CUR/DOX formulation, and 12.45% and 17.07% of the cells treated with the F-pHSM/DOX formulation. It is clear that the co-delivery of Pluronic L61 and curcumin significantly increased the cytotoxicity of DOX towards the MCF-7/ADR cells, which was also probably attributed to the synergistic MDR reversal effect of curcumin and Pluronic L61.

### Synergistic MDR reversal mechanism for the co-delivery of curcumin and Pluronic L61 unimers

#### Effects on the mitochondrial signalling pathways

The cytotoxicity assay revealed a comprehensive result that may stem from two features: acute necrosis and apoptosis arising from the combined curcumin and Pluronic L61 unimer therapy. Because both curcumin and Pluronics have been reported to target the mitochondria and have previously been used to treat MDR cancer cells, the mechanisms of the enhanced synergistic reversal effect induced by the co-delivery of curcumin and Pluronic L61 unimers were elucidated by examining the mitochondria-dependent apoptosis signalling pathways.

**Figure 9:**
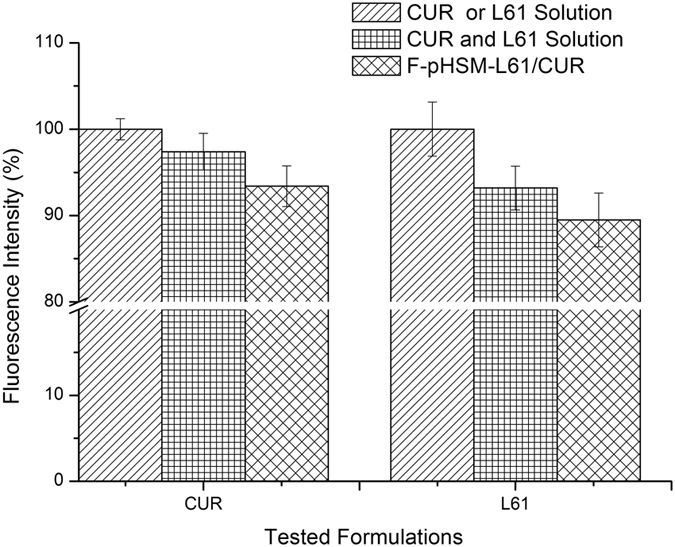
The accumulation of curcumin and Pluronic L61 in the mitochondria was measured by flow cytometry after the cells were incubated with the various drug formulations for 2 h. Each assay was performed in triplicate.

The co-delivery of curcumin and Pluronic L61 unimers to the mitochondria via the pH-sensitive micelles was quantified by measuring the fluorescence intensity using flow cytometry (Fig. 9). First, Pluronic L61 unimers were modified with RITC, which has strong red fluorescence. The fluorescence intensity of the curcumin and RITC-Pluronic L61 unimers in the mitochondria that had been treated with the curcumin solution and the RITC-Pluronic L61 unimer solution was set to 100%. MCF-7/ADR cells that had been treated with the F-pHSM-L61/CUR formulation showed a relative fluorescence intensity of 93.4% for curcumin and 89.5% for the RITC-Pluronic L61 unimers. Both curcumin and the RITC-Pluronic L61 unimers were effectively delivered to the cytoplasm by the F-pHSM-L61/CUR formulation and then selectively accumulated in the mitochondria.

**Figure 10:**
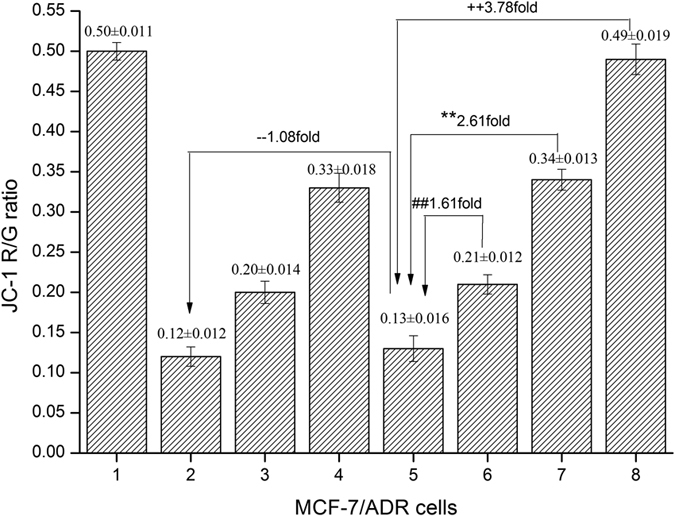
Effects of the blank mixed micelles (F-pHSM-L61/CUR, F-pHSM-L61, F-pHSM/CUR and F-pHSM) and the mixed Pluronic L61/CUR solution on the mitochondrial membrane potential of the MCF-7/ADR cells (mean ± S.D., n = 6). **P < 0.01: significantly different from the F-pHSM/CUR-treated cells, ^##^*P* < 0.01: significantly different from the F-pHSM-L61-treated cells, ^++^*P* < 0.01: significantly different from the F-pHSM-treated cells, ^−−^*P* > 0.05: not significantly different from cells treated with the mixture of Pluronic L61 and curcumin.

The mitochondrial membrane potential (MP) was determined using JC-1, which undergoes a reversible transformation from a monomer (green florescence) into an aggregate form (red florescence) when it binds to a membrane with a high MP. Mitochondrial depolarization (non-functional mitochondria) was indicated by a decrease in the ratio of the red/green fluorescence intensity. As shown in Fig. 10, compared to curcumin or the Pluronic L61 unimer solution, the mixed Pluronic L61/CUR solution significantly decreased the average JC-1 red/green fluorescence intensity ratio, indicating that curcumin or the Pluronic L61 unimers could exert synergistic mitochondrial depolarization effects. Among the tested micelles, the F-pHSM-L61/CUR formulation significantly decreased the average JC-1 red/green fluorescence intensity ratio in the MCF-7/ADR cells (R/G = 0.13 ± 0.016), which was not significantly different from that of the mixed Pluronic L61/CUR solution (R/G = 0.12 ± 0.012, *P* > 0.05). The F-pHSM-L61 formulation showed a moderate reduction of the fluorescence intensity ratio (R/G = 0.21 ± 0.012), followed by the F-pHSM/CUR formulation (R/G = 0.34 ± 0.013). The F-pHSM formulation had no effect on the MP (R/G = 0.49 ± 0.019) because it lacked Pluronic L61 and curcumin.

**Figure 11:**
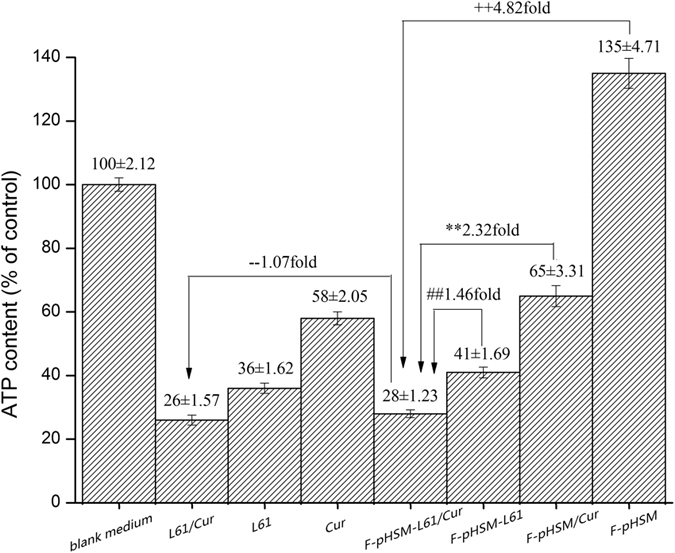
Effects of the blank mixed micelles (F-pHSM-L61/CUR, F-pHSM-L61, F-pHSM/CUR and F-pHSM) and the mixed Pluronic L61 unimers/CUR solution, the Pluronic L61 unimer solution, and the CUR solution on the intracellular ATP levels in MCF-7/ADR cells (mean ± S.D., n = 6). **P < 0.01: significantly different from the F-pHSM/CUR-treated cells, ^##^*P* < 0.01: significantly different from the F-pHSM-L61-treated cells, ^++^*P* < 0.01: significantly different from the F-pHSM-treated cells, ^−−^*P* > 0.05: not significantly different from cells treated with the mixture of Pluronic L61 unimers and curcumin.

The intracellular ATP levels were determined using luciferin/luciferase assays. The MCF-7/ADR cells treated with blank medium were used as controls, and their ATP levels were normalized to 100%. As shown in Fig. 11, the ATP levels in the MCF-7/ADR cells treated with the F-pHSM-L61/CUR formulation decreased to 28% of the normal level, which was not significantly different from the mixed Pluronic L61/CUR solution (26% of the normal level, *P* > 0.05). The F-pHSM-L61 and F-pHSM/CUR formulations decreased the ATP levels to 41% and 65% of the normal level, respectively. The ATP levels in the MDR cells treated with the F-pHSM formulation increased to approximately 135% due to energy-dependent endocytosis. The F-pHSM-L61/CUR formulation had a strong inhibitory effect on the ATP levels, suggesting that it can target the mitochondria and affect cell viability by inhibiting the respiratory chain^49^.

**Figure 12:**
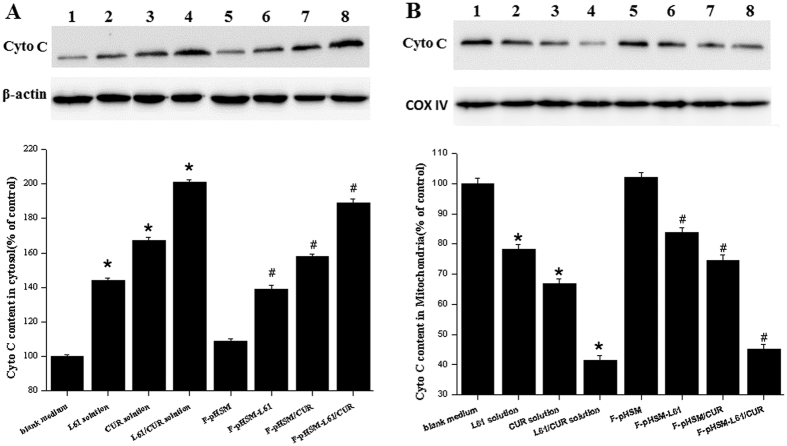
Levels of the cytochrome *c* protein in the cytosol (**A**) and mitochondria (**B**) of the MCF-7/ADR cells after a 24 h incubation with the different formulations (1: blank medium, 2: Pluronic L61 solution, 3: CUR solution, 4: the mixed Pluronic L61/CUR solution, 5: F-pHSM, 6: F-pHSM-L61, 7: F-pHSM/CUR and 8: F-pHSM-L61/CUR). *P < 0.05: significantly different from cells treated with the blank medium, ^#^*P* < 0.05: significantly different from the F-pHSM-treated cells. The blots were cropped and the full-length blots were included in the Supplementary Information.

The release of cytochrome *c* is an indicator of pro-apoptotic signalling^50^. Thus, the release of cytochrome *c* in the MCF-7/ADR cells was studied by Western blot analyses. The cytochrome *c* levels in the cytosol and mitochondria of the MCF-7/ADR cells that had been incubated with the different formulations are shown in Fig. 12A,B, respectively. Compared to the curcumin solution or the Pluronic L61 unimer solution, the mixed Pluronic L61/CUR solution significantly increased the cytochrome *c* content in the cytosol and decreased the cytochrome *c* content in the mitochondria, suggesting that curcumin and Pluronic L61 can synergistically induce mitochondrial dysfunction. Among the tested micelles, the F-pHSM-L61/CUR formulation significantly increased the cytochrome *c* content in the cytosol and decreased the cytochrome *c* content in the mitochondria. Notably, the F-pHSM-L61/CUR formulation induced effects on cytochrome *c* release that were comparable to the mixed Pluronic L61/CUR solution. Thus, the endosomal pH-triggered release and escape properties of the F-pHSM-L61/CUR formulation could effectively promote delivery of the curcumin and Pluronic L61 unimers into the cytosol, inducing mitochondrial dysfunction in the MCF-7/ADR cells.

**Figure 13:**
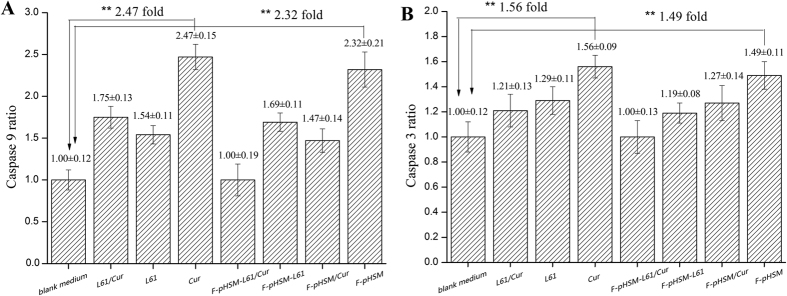
Caspase-9 (**A**) and caspase-3 (**B**) activities in the MCF-7/ADR cells after a 24 h incubation with the various drug formulations (blank medium, Pluronic L61 solution, CUR solution, mixed Pluronic L61/CUR solution, F-pHSM, F-pHSM-L61, F-pHSM/CUR and F-pHSM-L61/CUR). Each assay was repeated in triplicate. The data are presented as the mean ± S.D. (n = 3). **P < 0.01: significantly different from cells treated with the blank medium.

Caspase-9 and caspase-3 activities in MCF-7/ADR cells are presented in Fig. 13. Among the tested formulations, highly significant increases in the activities of both caspase-9 and caspase-3 were observed in the MCF-7/ADR cells after incubation with the mixed Pluronic L61 unimers/CUR solution. The activities of both caspase-9 and caspase-3 in the MCF-7/ADR cells treated with the F-pHSM-L61/CUR formulation were much higher than the corresponding activities after the addition of the other tested micelles.

In summary, the combination curcumin and Pluronic L61 unimer therapy significantly reduced mitochondrial MP and the ATP levels, increased the cytochrome *c* content in the cytosol and enhanced the activities of caspase-9 and caspase-3 compared to the monotherapy, indicating that curcumin and Pluronic L61 exerted a synergistic effect on mitochondria-dependent apoptosis signalling pathways^51^. Mitochondria exert both vital and lethal functions under physiological and pathological conditions^52^. Mitochondria are indispensable for energy production and are crucial regulators of the intrinsic pathway of apoptosis induced by intracellular stimuli, such as Ca^2+^ overload, the overproduction of reactive oxygen of species (ROS), the cleavage of apoptotic enzymes, and activation of members of the cysteine aspartic acid-specific protease (caspase) family^53^. As shown in previous studies, curcumin induces an increase in mitochondrial membrane permeability due to the formation of membrane pores. The permeability transition pores induce the collapse of the mitochondrial membrane potential and the inhibition of ATP synthesis, as well as the release of the apoptogenic factors and subsequent cell death^14^,^54^,^55^. As a mitochondrion-tropic molecule, Pluronic block copolymers target the mitochondria of MDR cancer cells and trigger cell apoptosis, as previously reported by our laboratory and other groups^37^. Pluronic block copolymers reduce the activity of the electron transport chain in the mitochondria^56^. There could be multiple mechanisms underlying the inhibitory activity of Pluronic copolymers in the mitochondria. The ability of Pluronic unimers to serve as K^+^ ionophores^57^ and uncouple oxidative phosphorylation likely contribute to their anti-metabolic effects^32^. Pluronic unimers may also directly inhibit the NADH dehydrogenase complex by interacting with the hydrophobic sites of this complex in the mitochondrial membrane^33^. In co-cultures, the enhanced MDR reversal activity of the F-pHSM-L61/CUR/DOX formulation might be because the two chemosensitizers could simultaneously reach the mitochondria without competition and then exert a synergistic MDR reversal effect through mitochondria-dependent apoptosis signalling pathways.

#### Effects on the expression and function of P-gp

**Figure 14:**
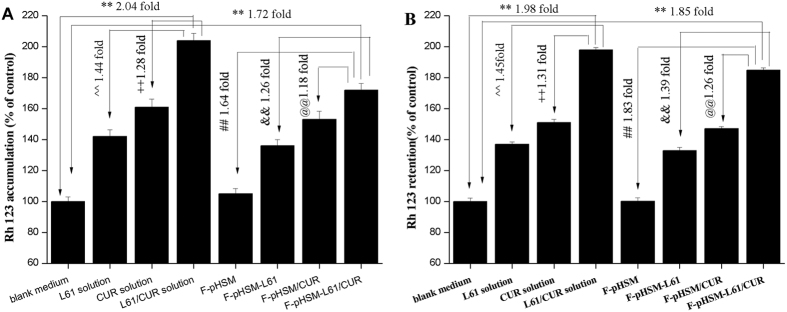
Effects of curcumin and Pluronic L61 unimers on Rh 123 accumulation in MCF-7/ADR cancer cells (**A**). Effects of curcumin and Pluronic L61 unimers on Rh 123 retention in MCF-7/ADR cancer cells (**B**). **P < 0.01: significantly different from cells treated with the blank medium, ^^P < 0.01: significantly different from cells treated with the L61 solution, ^++^P < 0.01: significantly different from cells treated with the CUR solution, ^##^P < 0.01: significantly different from the F-pHSM-treated cells, ^&&^P < 0.01: significantly different from the F-pHSM-L61-treated cells, ^@@^P < 0.01: significantly different from the F-PHSM/CUR-treated cells.

One of the major mechanisms of MDR is enhanced ability of tumour cells to actively efflux drugs, leading to a decrease in cellular drug accumulation below toxic levels. Active drug efflux is mediated by several ABC membrane transporters, and classical MDR is attributed to elevated P-gp expression. The most common method of reversing MDR is to administer non-toxic compounds or compounds with low toxicity that bind P-gp and block its transport function. Both curcumin and Pluronic copolymers were previously shown to effectively modulate the expression and function of P-gp in a wide variety of drug-resistant cells lines *in vitro*^58^,^59^,^60^,^61^. Rh 123 accumulation and efflux studies were performed to evaluate P-gp activity on the surface of viable cells because Rh 123 appears to be a sensitive indicator of P-gp activity. As shown in Fig. 14A, Rh 123 accumulation in MCF-7/ADR cancer cells was increased in response to treatment with all formulations, with the exception of F-pHSM. Compared to curcumin or Pluronic L61 unimer solutions, the mixed Pluronic L61/CUR solution significantly increased Rh 123 accumulation in the cells, indicating that curcumin or Pluronic L61 unimers exerted synergistic inhibition of P-gp. Moreover, there was no significant difference in Rh 123 accumulation between the cells treated with the F-pHSM-L61/CUR formulation and the mixed Pluronic L61/CUR solution. The effect of curcumin and the Pluronic L61 unimers on P-gp-mediated Rh 123 efflux in MCF-7/ADR cancer cells was also examined. As shown in Fig. 14B, both curcumin and the Pluronic L61 unimers induced a significant decrease in the amount of efflux Rh 123. The mixed Pluronic L61/CUR solution decreased Rh 123 efflux compared with the individual solutions. There was no significant difference in Rh 123 retention between the mixed Pluronic L61/CUR solution and the F-pHSM-L61/CUR formulation.

**Figure 15:**
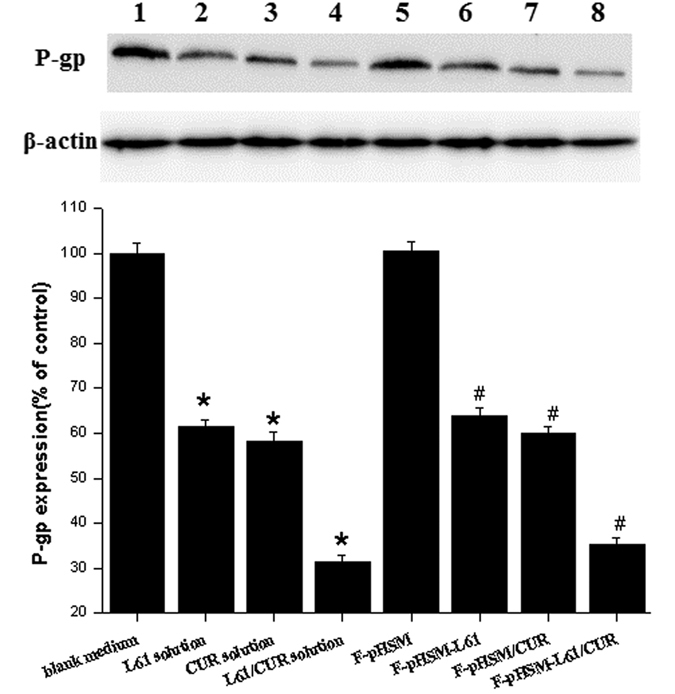
Effects of the various tested formulations on P-gp expression in MCF-7/ADR cells. (1) Control; (2) Pluronic L61 unimers; (3) Curcumin; (4) mixed Pluronic L61/CUR solution; (5) F-pHSM; (6) F-pHSM-L61/CUR; (7) F-pHSM-L61; (8) F-pHSM/CUR. *P < 0.05: significantly different from cells treated with the blank medium, ^#^*P* < 0.05: significantly different from the F-pHSM-treated cells. The blots were cropped and the full-length blots were included in the Supplementary Information.

MCF-7/ADR cells were treated with the mixed Pluronic L61/CUR solution, the Pluronic L61 solution, the CUR solution, or the F-pHSM-L61/CUR, F-pHSM-L61, F-pHSM/CUR or F-pHSM formulations to examine possible synergistic inhibitory effects on P-gp expression. As shown in Fig. 15, P-gp expression was decreased in MCF-7/ADR cells, with the exception of the cells treated with the F-pHSM formulation. The synergistic inhibition of P-gp expression is likely due to the reduced P-gp level observed in the cells treated with the mixed Pluronic L61/CUR solution compared with the cells treated with the individual solutions. The F-pHSM-L61/CUR formulation induced a relatively low P-gp expression in the cells treated with mixed Pluronic L61/CUR solution, and P-gp expression was reduced compared with the cells treated with both the F-pHSM-L61 and F-pHSM/CUR formulations, indicating that the intracellular co-delivery of Pluronic L61 and CUR synergistically inhibited P-gp.

Curcumin effectively modulates the expression and function of MDR proteins, including P-gp, MRP, LRP and BCRP, in a wide range of drug-resistant cell lines *in vitro*^10^,^58^,^59^,^60^. As reported by Anuchapreeda, curcumin decreased the level of immunoreactive P-gp protein observed in KB-V1 cells by down-regulating the MDR1 mRNA levels^62^. Although the mechanism by which curcumin down-regulated produced P-gp cannot be deduced from the present data, interestingly, the AP-1 transcription factor positively regulates MDR1 expression^63^. Curcumin has been reported to be a potent inhibitor of the AP-1 transcription factor^64^. Therefore, curcumin-mediated regulation of the MDR1 gene expression levels may be one mechanism to explain how curcumin inhibits MDR1 expression in the MDR cancer cells. In addition, P-gp inhibition by Pluronics involves at least three intracellular events: 1) inhibition of the P-gp ATPase, 2) a decrease in membrane microviscosity, and 3) a loss of the mitochondrial membrane potential, and a subsequent decrease in the ATP levels. However, no data have been reported on the modulation of P-gp expression by Pluronics in tumour cells. This study may be the first to report that Pluronics down-regulate the expression of a multi-drug-resistant transporter in mammalian cells. Pluronic block copolymers may prevent the development of MDR in cancer cells and “re-sensitize” resistant cancer cells to the level observed in the parental cells^61^,^65^. In this study, the intracellular co-delivery of curcumin and Pluronic L61 unimers significantly decreased the P-gp levels in MCF-7/ADR cancer cells, indicating that the two MDR reversal agents cooperated to regulate P-gp expression. However, additional studies are needed to elucidate the precise nature of the interaction between P-gp and curcumin and Pluronics.

### *In vivo* tumour targeting activity and anti-tumour efficacy of the pH-sensitive micelles

**Figure 16:**
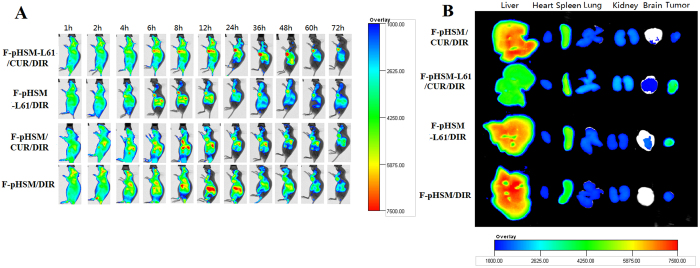
*In vivo* non-invasive images of the whole body of MCF-7/ADR tumour-bearing mice after i.v. injection of F-pHSM-L61/CUR/DiR, F-pHSM-L61/DiR, F-pHSM/CUR/DiR and F-pHSM/DiR were collected over time (**A**). *Ex vivo* optical images of tumours and organs from the MCF-7/ADR tumour-bearing mice, which were euthanized 48 h after the i.v. injection of F-pHSM-L61/CUR/DiR, F-pHSM-L61/DiR, F-pHSM/CUR/DiR and F-pHSM/DiR (**B**).

For effective drug-based cancer treatment, the drug must be specifically accumulated in tumours. The *in vivo* tumour targeting efficiency of the F-pHSM-L61/CUR micelles was evaluated in the MCF-7/ADR tumour-bearing mice using a non-invasive fluorescence imaging system with DiR as a probe. Real-time images of F-pHSM-L61/CUR/DiR micelles and control micelles (F-pHSM-L61/DiR, F-pHSM/CUR/DiR and F-pHSM/DiR formulations) are presented in Fig. 16A. The fluorescence intensity in the tumours from the F-pHSM-L61/CUR/DiR-injected animals was the highest among all of the tested micelle formulations. The F-pHSM-L61/CUR/DiR formulation increased the intra-tumour DiR concentration, indicating that the animals were protected by the Pluronic L61 unimers and curcumin through the synergistic reversal effect. According to the real-time imaging data, the fluorescence signals in the tumours increased for the first 24 h and were maintained for 72 h. The F-pHSM/DiR formulation was observed in highly perfused organs, such as the liver, due to the large amount of circulating blood that passes through these organs and the inevitable capture of the micelles by the reticuloendothelial system (RES). In contrast, the accumulation of the F-pHSM/DiR formulation in the tumour was quite low due to the absence of the MDR-reversing agents. The tumours were dissected at 72 h post-injection, and the fluorescence intensity was measured by *ex vivo* imaging (Fig. 16B). The fluorescence intensity of the F-pHSM-L61/CUR/DiR formulation in the tumours was much more intense than the intensities of the other tested micelles, which is comparable with the *in vivo* results observed at 72 h post-injection. The enhanced tumour targeting of the F-pHSM-L61/CUR formulation could be attributed to the synergistic tumour MDR reversal effects induced by the intracellular co-delivery of Pluronic L61 and CUR. Moreover, compared to the F-pHSM/CUR/DiR and F-pHSM/DiR formulations, both the F-pHSM-L61/CUR/DiR and F-pHSM-L61/DiR formulations significantly improved the fluorescence intensity in the brains of the MCF-7/ADR-xenografted mice. The enhanced brain accumulation might occur because Pluronic L61 inhibits the drug efflux transport systems in the blood-brain barrier (BBB)^66^. The BBB significantly restricts the delivery of both low molecular weight molecules and macromolecules to the brain. The low permeability of the BBB is largely attributed to the brain microvessel endothelial cells (BMVEC), which from tight extracellular junctions and have low pinocytic activity^67^. The specific efflux transporter systems that actively remove drugs and macromolecules from endothelial cells also limit the passage of selected drugs and macromolecules through the BBB. Pluronics were previously shown to enhance drug penetration of the BBB because they are able to block P-gp-related transport systems in the BBB. There are several mechanisms by which Pluronics inhibit P-gp in the BBB. First, the inhibitory effects of Pluronics on P-gp activity are at least partially attributable to alternations in the cellular ATP levels^33^,^68^. Second, Pluronics can increase membrane fluidity, which is attributed to alterations in the structure of the lipid bilayers as a result of the absorption of the block copolymer molecules on the membranes. Third, Pluronics exert a significant inhibitory effect on P-gp ATPase activity^69^.

**Figure 17:**
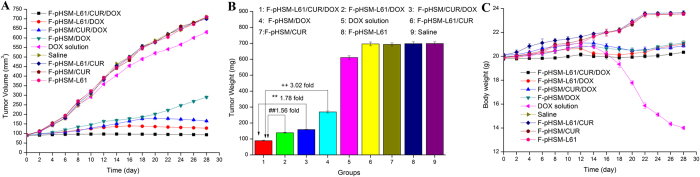
Changes in the tumour volumes in MCF-7/ADR tumour-bearing mice following the i.v. injection of saline, DOX solution, F-pHSM-L61/CUR/DOX, F-pHSM-L61/DOX, F-pHSM/CUR/DOX, F-pHSM/DOX, F-pHSM-L61/CUR, F-pHSM-L61 and F-pHSM/CUR (**A**). Weights of the tumours excised from the MCF-7/ADR tumour-bearing mice at the time of euthanasia (**B**). Variations in the body weights of the MCF-7/ADR tumour-bearing mice following the i.v. injection of saline, DOX solution, F-pHSM-L61/CUR/DOX, F-pHSM-L61/DOX, F-pHSM/CUR/DOX, F-pHSM/DOX, F-pHSM-L61/CUR, F-pHSM-L61 and F-pHSM/CUR (**C**) ^++^P < 0.01: significantly different from the F-pHSM/DOX-injected mice, **P < 0.01: significantly different from the F-pHSM/CUR/DOX-injected mice, ^##^P < 0.01: significantly different from the F-pHSM-L61/DOX-injected mice.

The changes in tumour volume as a function of time are shown in Fig. 17A. Blank micelles without DOX (F-pHSM-L61/CUR, F-pHSM/CUR and F-pHSM-L61) did not affect tumour development, which was in good correlation with the results of *in vitro* cytotoxicity assay. Upon treatment with free DOX, tumour growth was not obviously inhibited compared with that in the saline-treated group. In this case, the tumour growth inhibition (TGI) was only 12.67%, indicating that the chemotherapy failed to affect the drug-resistant cancer. However, compared to the DOX solution after intravenous injection, all of the tested DOX-loaded micelles showed significant therapeutic effects on suppressing tumour growth. Compared to other micelles, the F-pHSM-L61/CUR/DOX formulation induced the most pronounced tumour growth inhibition, as indicated by the high TGI of 99.37%. The final average tumour weight of the F-pHSM-L61/CUR/DOX-treated mice was 89.33 ± 3.14 mg, which was significantly reduced compared to the tumour weights of the mice treated with the F-pHSM-L61/DOX (139.54 ± 2.48 mg), F-pHSM/CUR/DOX (159.27 ± 3.01 mg) and F-pHSM/DOX (269.89 ± 6.71 mg) formulations (Fig. 17B). The superior therapeutic efficacy of the F-pHSM-L61/CUR/DOX formulation is attributed to the synergistic MDR reversal effect induced by co-delivery of curcumin and the Pluronic L61 unimers. Changes in body weight during the treatment period are shown in Fig. 17C. The F-pHSM-L61/CUR/DOX-, F-pHSM-L61/DOX- and F-pHSM/CUR/DOX-treated groups did not display obvious body weight fluctuations. Because the tumour weights were significantly reduced compared to those of the control group, the micelles were more beneficial to the health of the mice than free DOX. The intracellular co-delivery of different MDR-modulating agents via pH-sensitive micelles is a promising approach for reversing tumour MDR.

**Figure 18:**
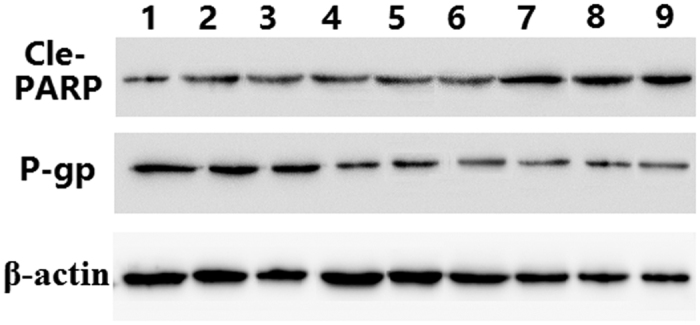
Expression of P-gp and Cle-PARP in tumour tissue form mice after treatment with different formulations: 1. Saline; 2. DOX solution; 3. F-pHSM; 4. F-pHSM/CUR; 5. F-pHSM-L61; 6. F-pHSM-L61/CUR; 7. F-pHSM/CUR/DOX; 8. F-pHSM-L61/DOX; 9. F-pHSM-L61/CUR/DOX.

Then we further determined the levels of P-gp in tumour tissue. Western blotting results of P-gp levels in tumour tissue were in accordance with the *in vitro* findings. As shown in Fig. 18, P-gp expression was decreased in MCF-7/ADR cells, with the exception of the cells treated with the Saline, DOX solution and F-pHSM/DOX formulation. Both the F-pHSM-L61/CUR and F-pHSM-L61/CUR/DOX could significantly reduce P-gp level observed in the tumour tissue due to the synergistic P-gp expression inhibition of L61 and CUR compared with the tumour treated with the individual formulations. To confirm whether the enhanced anti-tumour effect of F-pHSM-L61/CUR/DOX *in vivo* was related to the pro-apoptosis activity, apoptosis related protein Cle-PARP was measured by western blotting. As shown in Fig. 18, compared with that of control groups (saline, F-pHSM-L61/CUR, F-pHSM-L61 and F-pHSM/CUR), the bands in groups administrated with free drugs, F-pHSM/CUR/DOX, F-pHSM-L61/DOX and F-pHSM-L61/CUR/DOX were more evident. In particular, F-pHSM-L61/CUR/DOX possessed the highest expression. Therefore, the expression of apoptotic protein in the decreasing order of F-pHSM-L61/CUR/DOX > F-pHSM-L61/DOX > F-pHSM/CUR/DOX > F-pHSM /DOX > DOX solution coincident with the tumour inhibition results suggested that the enhanced antitumor efficacy of F-pHSM-L61/CUR/DOX was related to the activation of cell apoptosis process and mitochondria-mediated cell death causing by the synergistic reversal effect of curcumin and L61.

Meanwhile, alanine transaminase (ALT), aspartate transaminase (AST), creatine kinase (CK) and lactate dehydrogenase (LDH) levels of the blood samples obtained from the mice with and without the treatment of the micelles were also measured to study the toxicity of the micelles to the liver and heart. DOX has well established that the most common side effect is cardiotoxicity, while the cumulative and irreversible cardiotoxicity is the major limiting factor of DOX in the clinical setting^70^,^71^,^72^. As listed in Table 2, treatment with DOX solution could significantly increase the CK and LDH levels of the blood samples, indicating that the significant damage to the heart function at the tested dose. While, treatment with the DOX-loaded micelles did not elevate the levels of CK and LDH, indicating that the micelles did not cause significant damage to the heart functions at the tested dose. In addition, DOX-loaded micelles also did not elevate the levels of ALT and AST, indicating combined therapy did not have significant liver injury.

## Conclusions

In summary, the synergistic MDR reversal effect induced by curcumin and the Pluronic L61 unimers was evaluated using a system designed for intracellular co-delivery with pH-sensitive micelles. The micellar delivery system included a copolymer of PHis-PLA-PEG-PLA-PHis and Pluronic F127 that was partially conjugated with folate to ensure intracellular co-delivery via endosomal pH-triggered drug release and copolymer-facilitated endosomal escape. Compared with the delivery of a single drug, the intracellular co-delivery of curcumin and the Pluronic L61 unimers resulted in increased *in vitro* cytotoxicity, cellular uptake and cell apoptosis in MCF-7/ADR cells and inhibited tumour growth in mice *in vivo*. Mechanistically, both the Pluronic L61 unimers and curcumin were selectively accumulated in the mitochondria and synergistically reversed MDR by inhibiting mitochondrial signalling pathways and decreasing the expression and function of P-gp in the MCF-7/ADR cells to reduce drug efflux. Intracellular co-delivery of different MDR-modulating agents is as a promising approach to reverse MDR.

## Additional Information

**How to cite this article**: Hong, W. *et al*. pH-sensitive micelles for the intracellular co-delivery of curcumin and Pluronic L61 unimers for synergistic reversal effect of multidrug resistance. *Sci. Rep.* **7**, 42465; doi: 10.1038/srep42465 (2017).

**Publisher's note:** Springer Nature remains neutral with regard to jurisdictional claims in published maps and institutional affiliations.

## Supplementary Material

Supplementary Information

## Figures and Tables

**Figure 1:**
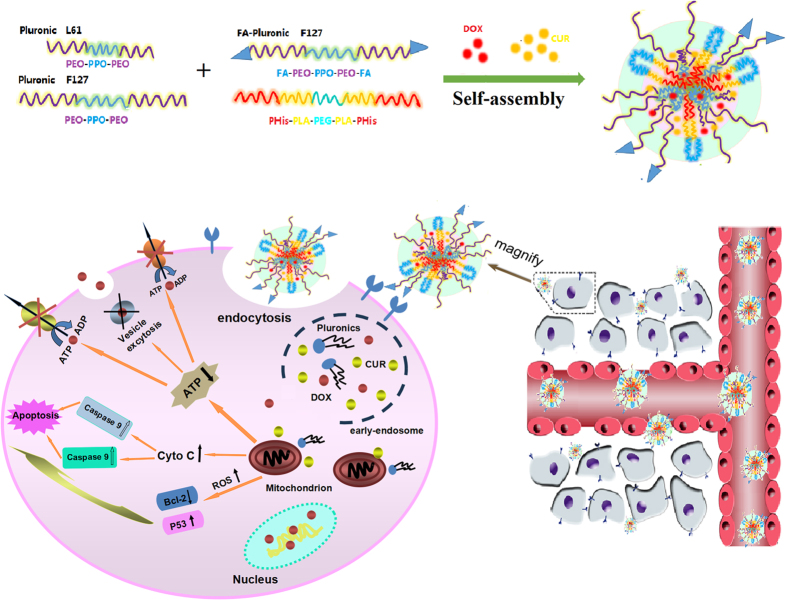
Schematic illustration of the design and proposed mechanism of F-pHSM-L61/CUR/DOX to exert synergistic MDR reversal effect.

**Table 1:** Physicochemical characterization of DOX-loaded polymeric mixed micelles (n = 3).

Formulations	Particle Size (nm)	ξ potential (mv)	PDI	DOX	
				DL%	EE%
F-pHSM-L61/CUR/DOX	228.7 ± 13.6	−6.09 ± 0.18	0.089 ± 0.005	4.27 ± 0.12	85.4 ± 1.18
F-pHSM/CUR/DOX	225.6 ± 12.1	−6.11 ± 0.09	0.067 ± 0.007	4.31 ± 0.13	86.2 ± 1.32
F-pHSM-L61/DOX	192.0 ± 18.9	−5.68 ± 0.11	0.091 ± 0.004	4.51 ± 0.12	90.3 ± 1.23
F-pHSM/DOX	188.0 ± 15.6	−5.94 ± 0.13	0.084 ± 0.006	4.54 ± 0.11	90.7 ± 1.14

**Table 2:** Effect of micelles on the liver and heart functions (n = 6).

Treatment	ALT (U/L)	AST (U/L)	CK (U/L)	LDH (U/L)
Saline	30.78 ± 5.89	49.71 ± 10.52	626 ± 58	489 ± 27
DOX Solution	31.23 ± 4.27	50.12 ± 7.84	1089 ± 124	937 ± 56
F-pHSM-L61/CUR	29.33 ± 4.12	49.01 ± 9.98	664 ± 37	491 ± 23
F-pHSM /CUR	30.54 ± 3.21	51.27 ± 10.45	659 ± 49	474 ± 19
F-pHSM-L61	29.48 ± 2.12	50.81 ± 7.77	637 ± 51	493 ± 28
F-pHSM-L61/CUR/DOX	31.55 ± 2.69	49.96 ± 8.12	612 ± 37	477 ± 33
F-pHSM/CUR/DOX	29.97 ± 2.59	50.27 ± 7.23	644 ± 42	490 ± 21
F-pHSM-L61/DOX	30.12 ± 4.31	49.23 ± 5.12	656 ± 59	499 ± 17
F-pHSM /DOX	30.81 ± 3.22	50.33 ± 6.89	671 ± 29	482 ± 22
