# Author Correction: pH-sensitive micelles for the intracellular co-delivery of curcumin and Pluronic L61 unimers for synergistic reversal effect of multidrug resistance

**DOI:** 10.1038/s41598-021-97676-8

**Published:** 2021-09-06

**Authors:** Wei Hong, Hong Shi, Mingxi Qiao, Zehui Zhang, Wenting Yang, Lingying Dong, Fucheng Xie, Chunpeng Zhao, Li Kang

**Affiliations:** 1 Key Laboratory of Zoonosis of Liaoning Province, College of Animal Science and Veterinary Medicine, Shenyang Agricultural University, Dongling Road 120, Shenyang, 110866 Liaoning Province People’s Republic of China; 2 School of Pharmacy, China Pharmaceutical University, Longmian Avenue 639, Jiangning District, Nanjing, 211198 People’s Republic of China; 3 School of Pharmacy, Shenyang Pharmaceutical University, Wenhua Road 103, Shenyang, 110016 Liaoning Province People’s Republic of China

Correction to: *Scientific Reports* 10.1038/srep42465, published online 14 February 2017

This Article contains errors.

The image used for Figure 2 is incorrect. The correct Figure 2 and its accompanying legend appear below.

**Figure 2:**
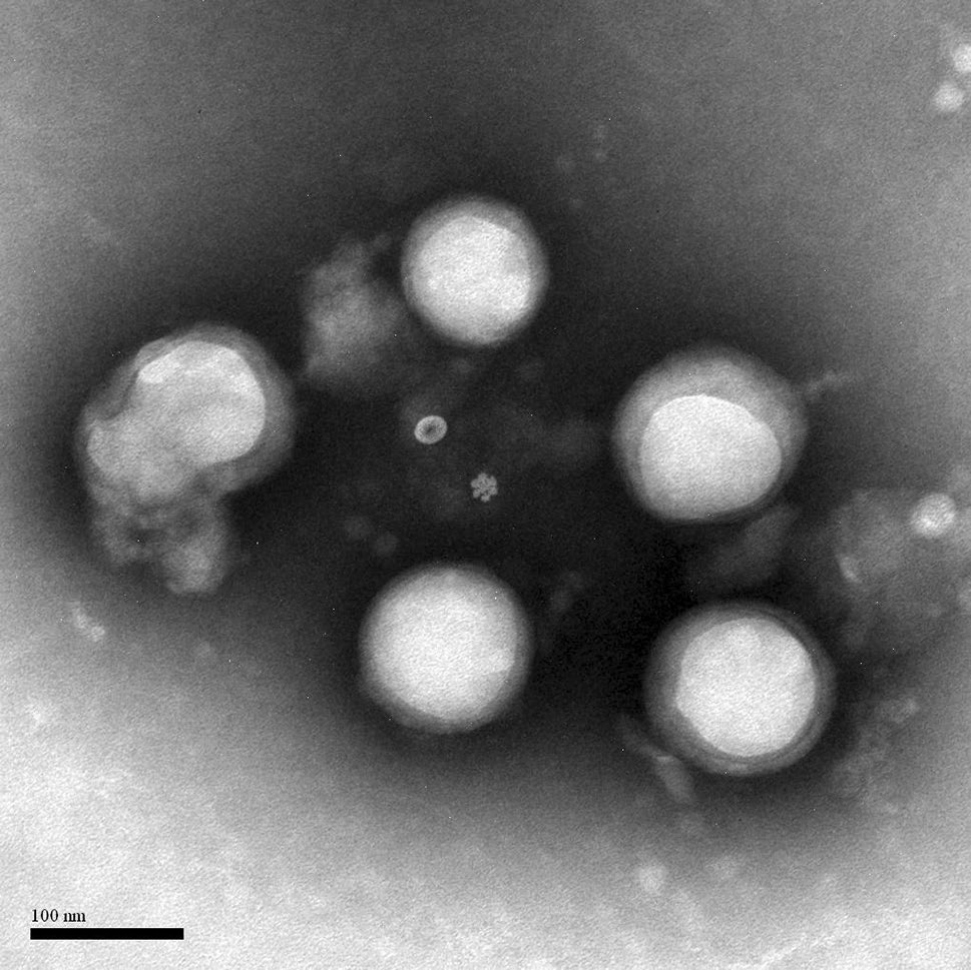
TEM images of F-pHSM-L61/CUR/DOX.

Consequently, the particle size and zeta potential reported in Table 1 is incorrect. A corrected version of Table 1 appears below.

**Table 1:** Physicochemical characterization of DOX-loaded polymeric mixed micelles (n = 3).

Formulations	Particle size (nm)	ξ potential (mv)	PDI	DOX	
				DL%	EE%
F-pHSM-L61/CUR/DOX	221.3 ± 5.3	− 6.18 ± 0.21	0.193 ± 0.028	4.03 ± 0.12	80.6 ± 1.22
F-pHSM/CUR/DOX	218.7 ± 4.8	− 6.23 ± 0.17	0.152 ± 0.022	4.05 ± 0.16	81.1 ± 1.56
F-pHSM-L61/DOX	193.5 ± 3.4	− 5.72 ± 0.23	0.129 ± 0.031	4.49 ± 0.12	90.0 ± 1.17
F-pHSM/DOX	179.1 ± 3.1	− 5.98 ± 0.19	0.134± 0.015	4.57 ± 0.12	91.4 ± 1.21

In Figure 8, the panels for 8A and 8B are incorrect. The correct Figure 8 and its accompanying legend appear below.

**Figure 8:**
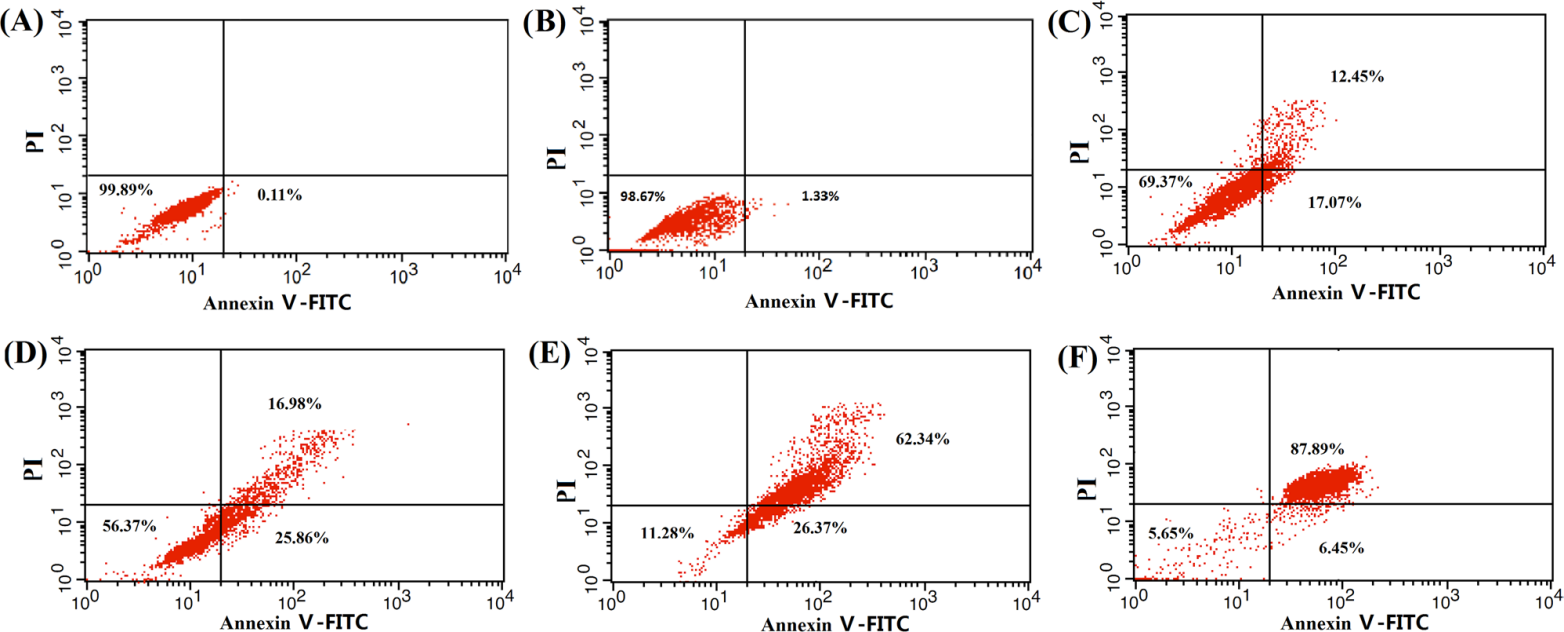
Synergistic effect of micelle-encapsulated Pluronic L61 unimers and curcumin on apoptosis of MCF-7/ADR cells treated with saline (**A**), F-pHSM (**B**), F-pHSM/DOX (**C**), F-pHSM/CUR/DOX (**D**), F-pHSM-L61/DOX (**E**) and F-pHSM-L61/CUR/DOX (**F**).

In Figure 15, the blots for beta-actin and P-gp were inadvertently duplicated from Figure 12, which affected the quantification. A corrected version of Figure 15 and its accompanying legend appear below.

**Figure 15:**
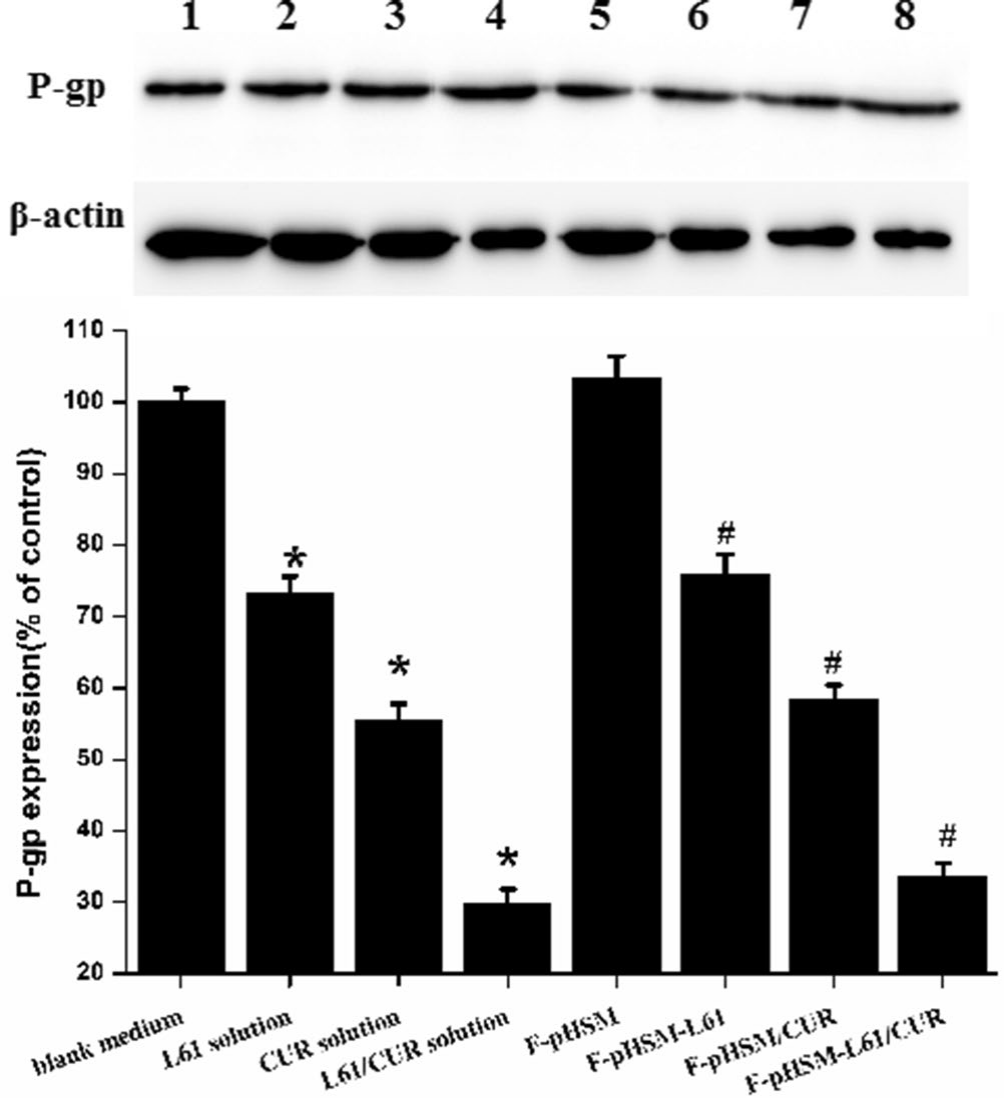
(1) Control; (2) Pluronic L61 unimers; (3) Curcumin; (4) mixed Pluronic L61/CUR solution; (5) F-pHSM; (6) F-pHSM-L61/CUR; (7) F-pHSM-L61; (8) F-pHSM/CUR. *P < 0.05: significantly different from cells treated with the blank medium, #P < 0.05: significantly different from the F-pHSM-treated cells. The blots were cropped and the full-length blots were included in the Supplementary Information.

In Figure 18, the blots provided are incorrect. A corrected version of Figure 18 is presented below, and a quantitative analysis of P-gp and Cle-PARP expression has been added. The Figure legend has been revised accordingly.

Consequently, the text in the Results and Discussion,

“As shown in Fig. 18, P-gp expression was decreased in MCF-7/ADR cells, with the exception of the cells treated with the Saline, DOX solution and F-pHSM/DOX formulation. Both the F-pHSM-L61/CUR and F-pHSM-L61/CUR/DOX could significantly reduce P-gp level observed in the tumor tissue due to the synergistic P-gp expression inhibition of L61 and CUR compared with the tumor treated with the individual formulations. To confirm whether the enhanced anti-tumor effect of F-pHSM-L61/CUR/DOX in vivo was related to the pro-apoptosis activity, apoptosis related protein Cle-PARP was measured by western blotting. As shown in Fig. 18, compared with that of control groups (saline, F-pHSM-L61/CUR, F-pHSM-L61 and F-pHSM/CUR), the bands in groups administrated with free drugs, F-pHSM/CUR/DOX, F-pHSM-L61/DOX and F-pHSM-L61/CUR/DOX were more evident.

should read:

“As shown in Fig. 18, all treatments conferred a reduction in P-gp level. Among the tested groups, F-pHSM-L61/CUR/DOX significantly reduced the P-gp level observed in the tumor tissue, which might be due to the synergistic P-gp expression inhibition effect of L61 and CUR. Additionally, F-pHSM-L61/CUR showed a comparable inhibition effect as F-pHSM-L61/CUR/DOX, which is consistent with there being a strong synergistic action between L61 and CUR. Furthermore, apoptosis related protein Cle-PARP was measured by western blotting to investigate whether the enhanced anti-tumor effect of F-pHSM-L61/CUR/DOX in vivo was related to the pro-apoptosis activity. As shown in Fig. 18C, compared with that of control groups (saline, F-pHSM-L61/CUR, F-pHSM-L61 and F-pHSM/CUR), the effect of groups administrated with free drugs, F-pHSM/DOX, F-pHSM/CUR/DOX, F-pHSM-L61/DOX and F-pHSM-L61/CUR/DOX were more evident.”

**Figure 18:**
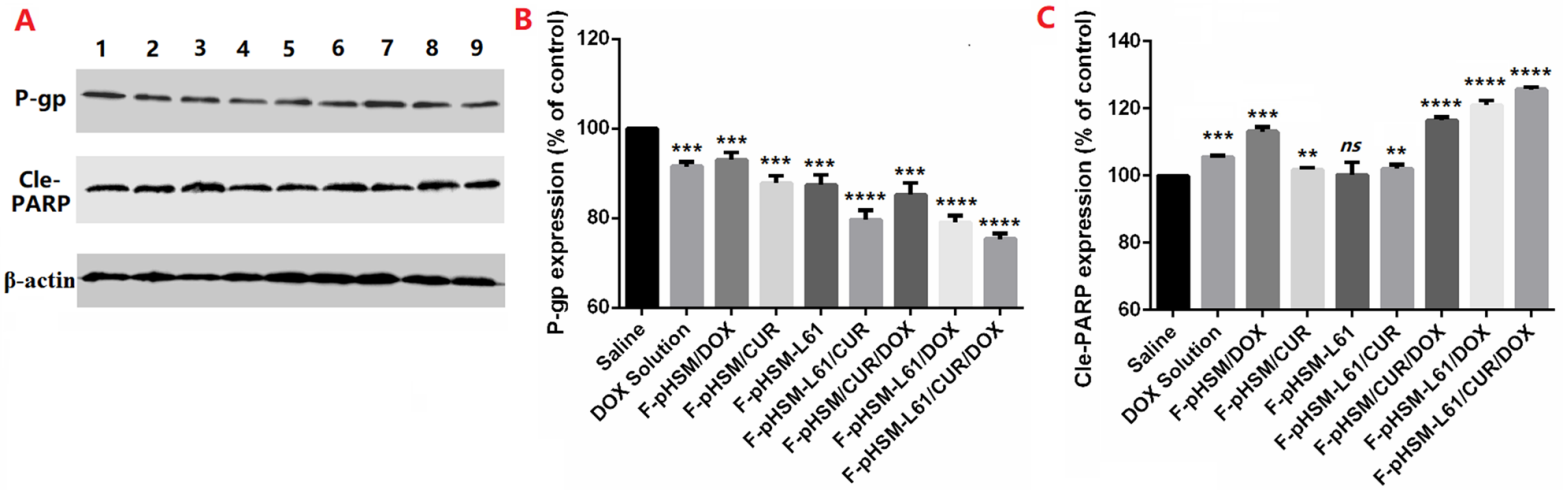
Expression of P-gp and Cle-PARP in tumor tissue from mice after treatment with different formulations (**A**). Quantitative analysis of the expression of P-gp (**B**) and Cle-PARP (**C**) in tumor tissue from mice after treatment with different formulations: 1. Saline; 2. DOX solution; 3. F-pHSM/DOX; 4. F-pHSM/CUR; 5. F-pHSM-L61; 6. F-pHSM-L61/CUR; 7. F-pHSM/CUR/DOX; 8. F-pHSM-L61/DOX; 9. F-pHSM-L61/CUR/DOX. Statistical significance was defined as *P <0.05, **P <0.01, ***P <0.001 and ****P <0.0001. One data-point for F-pHSM-L61/CUR was omitted, as it was considered an outlier (more than 3 standard deviations).

As a result of the above, the original blots provided in the Supplementary Information file for Figure 15 and 18 are incorrect. The correct Supplementary Information File is provided below.

## Supplementary Information

Supplementary Information.

