# Supplementary material for: Author Correction: pH-sensitive micelles for the intracellular co-delivery of curcumin and Pluronic L61 unimers for synergistic reversal effect of multidrug resistance
Source: Sci Rep. 2021 Sep 6;11:18061. doi: 10.1038/s41598-021-97676-8 (PMC8421502; doi:10.1038/s41598-021-97676-8)

***Supplementary Information for***

pH sensitive micelles for intracellular co-delivery of Curcumin and Pluronic L61 unimers for synergistic reversal effect of multidrug resistance

Wei Hong^a*^, Hong Shi^a,b^, Mingxi Qiao^c^, Zehui Zhang^a^, Wenting Yang^a^, Lingying Dong^a^, Fucheng Xie^a^, Chunpeng Zhao^a^, and Li Kang^a^

*^a^ Key laboratory of Zoonosis of Liaoning Province, College of Animal Science and Veterinary Medicine, Shenyang Agricultural University, Dongling Road 120, Shenyang, Liaoning Province, 110866, P.R. China*

*^b^ School of Pharmacy, China Pharmaceutical University, #639 Longmian Avenue, Jiangning District, Nanjing, 211198, P.R. China*

*^c^ School of Pharmacy, Shenyang Pharmaceutical University, Wenhua Road 103, Shenyang, Liaoning Province, 110016, P.R. China*

*^*^Corresponding author . Tel: +86-24-88487156;*

*Fax:+86-24-88487156;*

*E-mail address: hongwei_sy@163.com*

**Experimental section**

**The optimum loading content of CUR on the MDR reversal**

The optimum loading content of curcumin used in the tested micelles was determined through MTT method. Firstly, the MCF-7/ADR cancer cells were seeded at the density of 5×10^3^ cells per well in 96-well plates and incubated for 24 h. Then the growth medium was replaced with fresh medium containing an indicated CUR concentration (0.25, 0.5, 1, 2, 4, 8, 16, 32 and 64 μg/mL), respectively. Control wells were treated with equivalent volume of CUR-free medium. The cells were incubated at 37 ºC for 48 h. After incubation, the wells were rinsed with PBS, and then MTT solution (5 mg/mL) was added to each well and the plate was incubated for 4 h. At last, the medium was removed completely and 150 µL of dimethyl sulphoxide (DMSO) was added to each well to dissolve purple formazan crystals. The absorbance was measured with at 570 nm using a multifunctional microplate reader (Tecan, Austria). The IC_50_ values were calculated using nonlinear regression analysis. Secondly, another batch of MCF-7/ADR cancer cells were seeded at the density of 5×10^3^ cells per well in 96-well plates and incubated for 24 h. Then the growth medium was replaced with fresh medium containing an indicated CUR concentration (below IC_50_ values), respectively. Subsequently, cell viability was also measured using MTT method. The mean percentage of cell survival relative to that of untreated cells was estimated from data of six individual experiments, and all data were expressed as the mean±SD. The cell viability higher than 90% indicated very low cytotoxicity.

**The optimum loading content of Pluronic L61 on the MDR reversal**

The optimum loading content of Pluronic L61 used in the tested micelles was first determined through MTT method. Briefly, the MCF-7/ADR cancer cells were seeded at the density of 5×10^3^ cells per well in 96-well plates and incubated for 24 h. Then the cells were exposed to a serious of DOX concentration of F-pHSM-L61/DOX with different weight ratios of Pluronic L61 (3×10^-6^, 6×10^-6^, 3×10^-5^, 6×10^-5^, 3×10^-4^, 6×10^-4^, 3×10^-3^, 6×10^-3^, 3×10^-2^ and 0.3 wt%), respectively. Control wells were treated with equivalent volume of DOX-free medium. The cells were incubated at 37 ºC for 48 h. After incubation, the wells were rinsed with PBS, and then MTT solution (5 mg/mL) was added to each well and the plate was incubated for 4 h. At last, the medium was removed completely and 150 µL of dimethyl sulphoxide (DMSO) was added to each well to dissolve purple formazan crystals. The absorbance was measured with at 570 nm using a multifunctional microplate reader (Tecan, Austria). The IC_50_ values were calculated using nonlinear regression analysis and the MDR reversal effect was assessed by quantifying the IC_50_ values of the tested formulations.

Then, the optimum loading content of Pluronic L61 used in the tested micelles was further detected using flow cytometry. Briefly, the MCF-7/ADR cancer cells were seeded at the density of 1×10^5^ cells/well in 6-well plates and incubated for 24 h to allow cell attachment. After 24 h, the medium was replaced with cell culture medium containing F-pHSM-L61/DOX with different weight ratios of Pluronic L61 (3×10^-6^, 6×10^-6^, 3×10^-5^, 6×10^-5^, 3×10^-4^, 6×10^-4^, 3×10^-3^, 6×10^-3^, 3×10^-2^ and 0.3 wt%), respectively. After 4 h incubation, the cells were washed three times with PBS. The cells were then harvested by trypsinization, centrifuged at 1000 rpm for 5 min, re-suspended in 500 µL of PBS medium and analyzed using a BD FACS Caliber flow cytometer (FACSCAN, Becton Dickinson, San Jose, CA, USA).

**Results**

**The optimum loading content of CUR on the MDR reversal**

The optimum loading content of CUR was evaluated by MTT method. The IC_50_ value of CUR solution was 12.84±0.36 µg/mL. In order to exclude the cytotoxicity of CUR solution, we should use a dosage that does not significantly kill of retard cell growth. The MCF-7/ADR cancer cells were treated with different concentrations of curcumin (1, 2, 4, 6, 8, 10 and 12 µg/mL), respectively and the cell viability was summarized in Table S1. As shown in Table S1, the cell viability of MCF-7/ADR cancer cells decreased with the increasing of curcumin concentrations. When the concentration of curcumin was higher than 6 µg/mL, the cell viability of MCF-7/ADR cancer cells was lower than 90%. Therefore, the 4 µg/mL of curcumin (1×10^-2^ wt%) was selected for the following studies.

Table S1 The cell viability of MCF-7/ADR cancer cells treated with different concentrations of curcumin (n=6)

| Con.( µg/mL) | 1 | 2 | 4 | 6 | 8 | 10 | 12 |
| --- | --- | --- | --- | --- | --- | --- | --- |
| Cell viability (%) | 99.26±0.65 | 97.01±0.37 | 95.11±0.29 | 85.15±0.73 | 76.12±0.32 | 69.23±0.48 | 57.84±0.89 |

**The effect of loading content of Pluronic L61 on the MDR reversal**

The effect of loading content of Pluronic L61 unimers in F-pHSM-L61/DOX on the cytotoxicity and cellular uptake was also evaluated using MTT method and flow cytometry, respectively (Fig. S1A and Fig. S1B). The IC_50_ value decreased with the increasing of Pluronic L61 loading content until the loading content reached 3×10^-4^ wt%. When the loading content was higher than 3×10^-4^ wt%, the cytotoxicity of DOX against MCF-7/ADR cells tended to dramatically decrease and then leveled off. Similar results were obtained in the celluar uptake study using flow cytometry. The maximum celluar uptake occurred when the loading content of Pluronic L61 was 3×10^-4^ wt%. Further increasing the loading content of Pluronic L61, the cellular uptake first decreased and then leveled off (Fig. S1B).


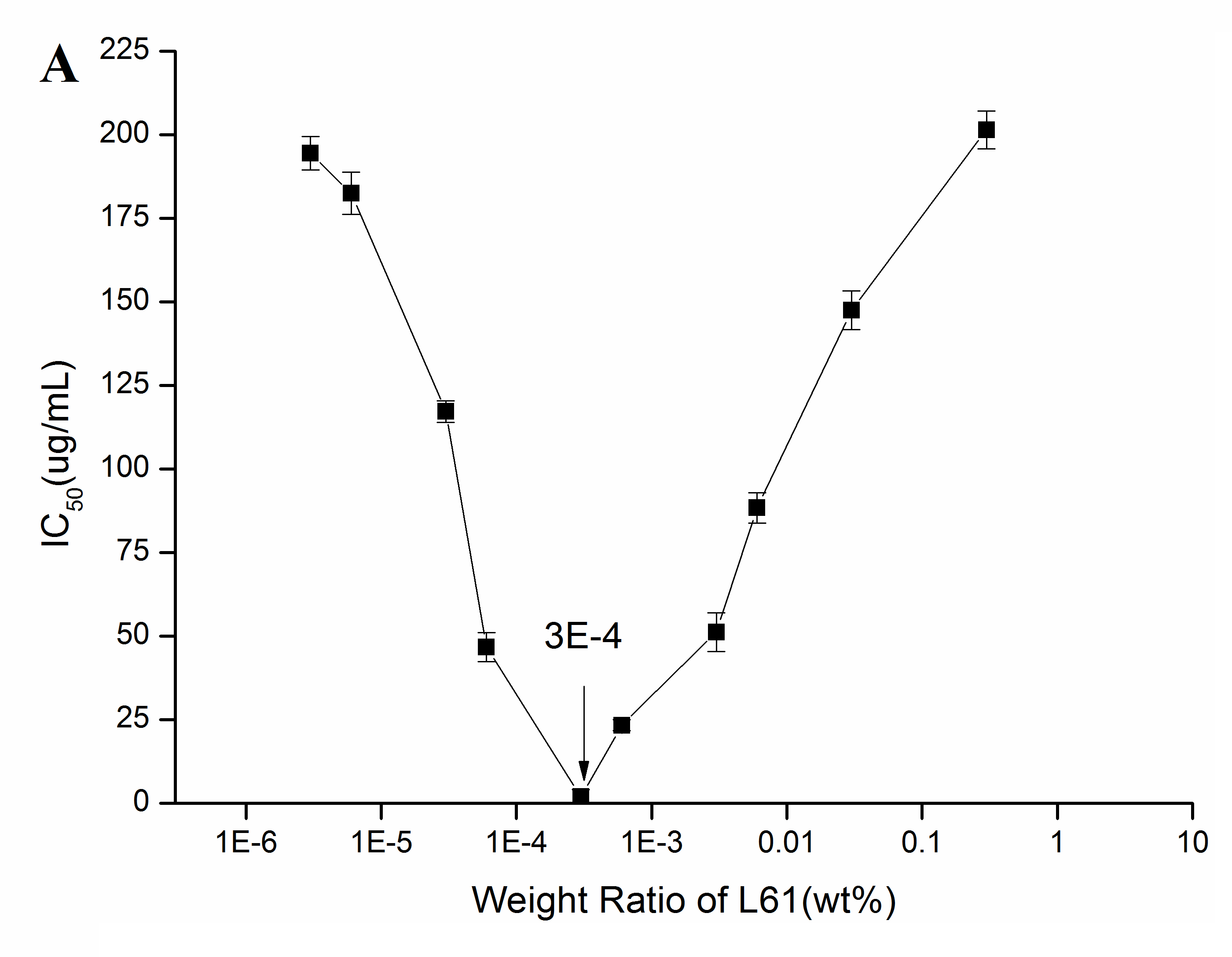


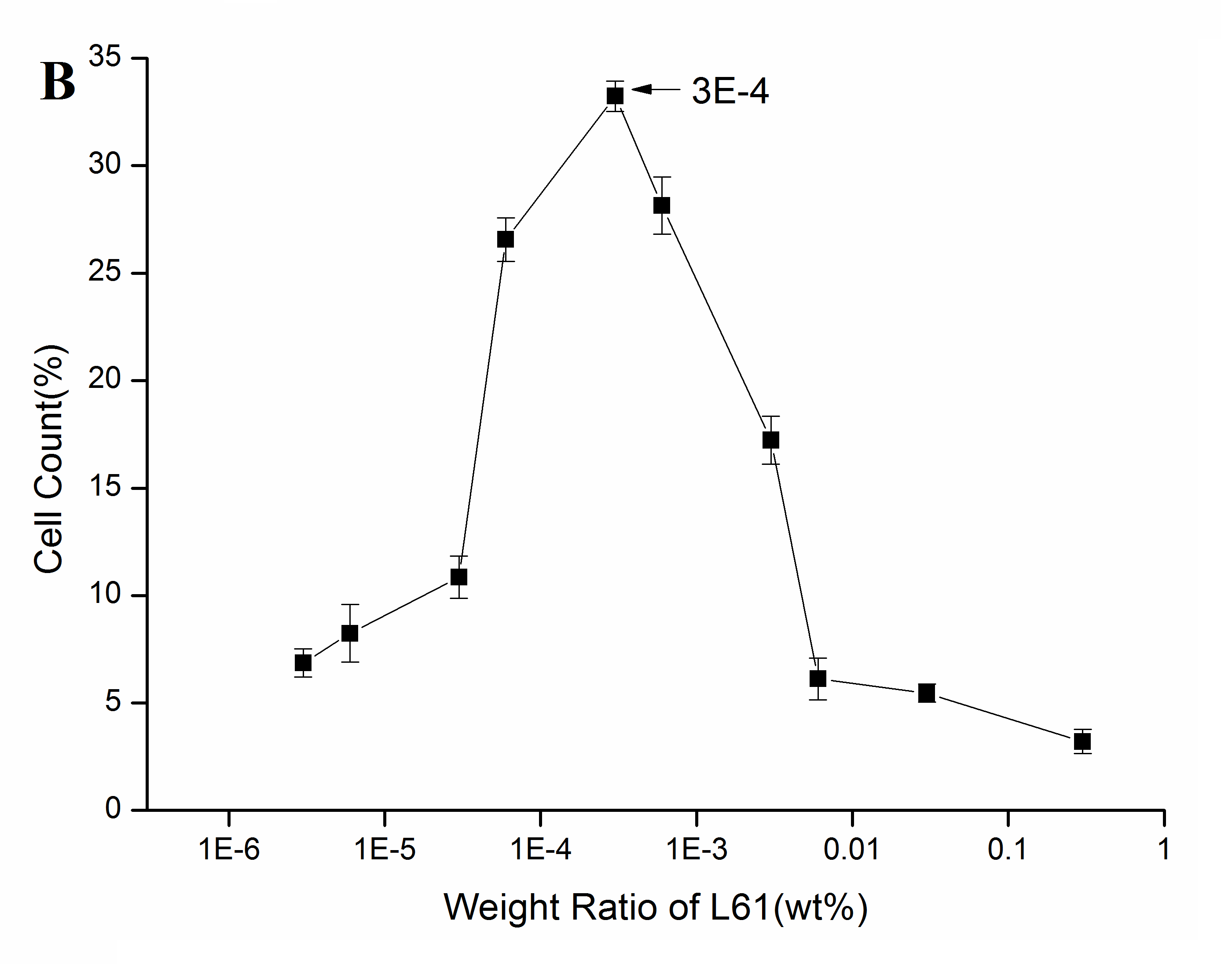


Fig. S1 In *vitro* cytotoxicity of the F-pHSM-L61/CUR/DOX with different loading content of Pluronic L61 against MCF-7/ADR cells (A) and flow cytometry analysis of the F-pHSM-L61/CUR/DOX with different loading content of Pluronic L61 uptaken by MCF-7/ADR cells (B) (mean ± SD, n=6).

**The full-length blots of Fig. 12A and 12 B.**


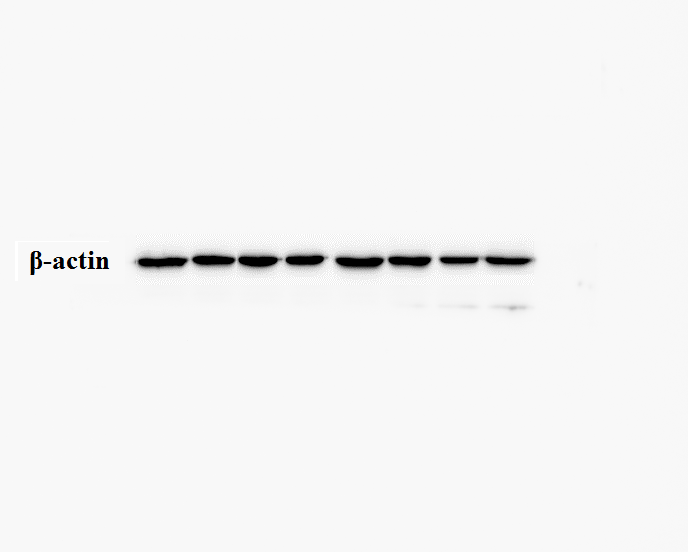

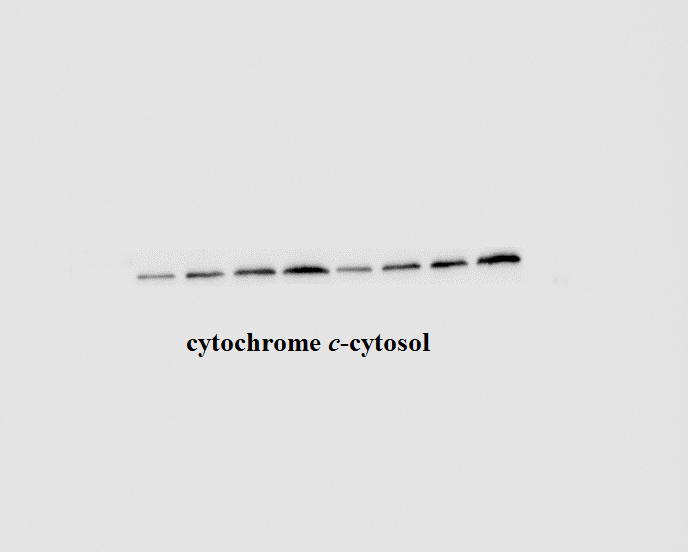

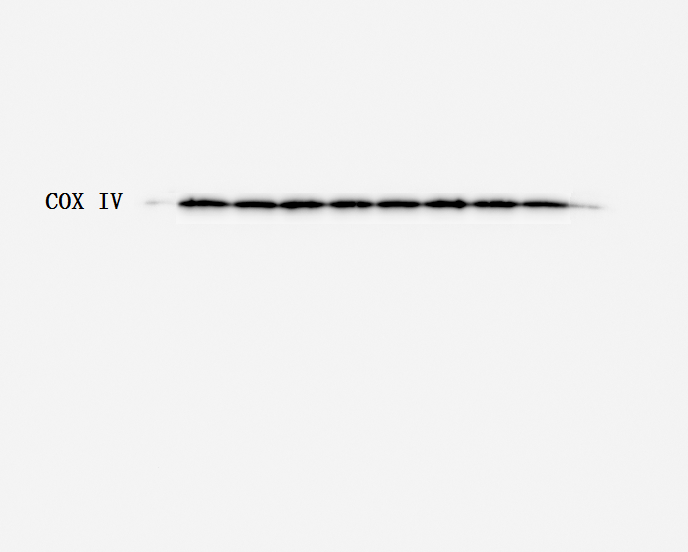

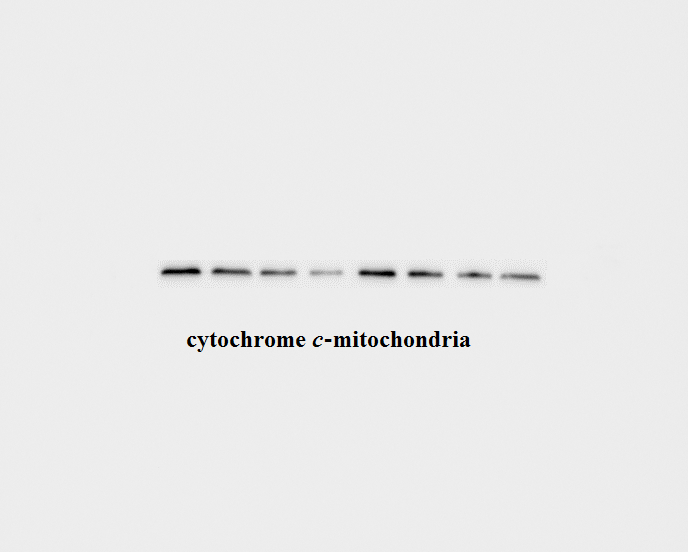


**The full-length blots of Fig. 15.**


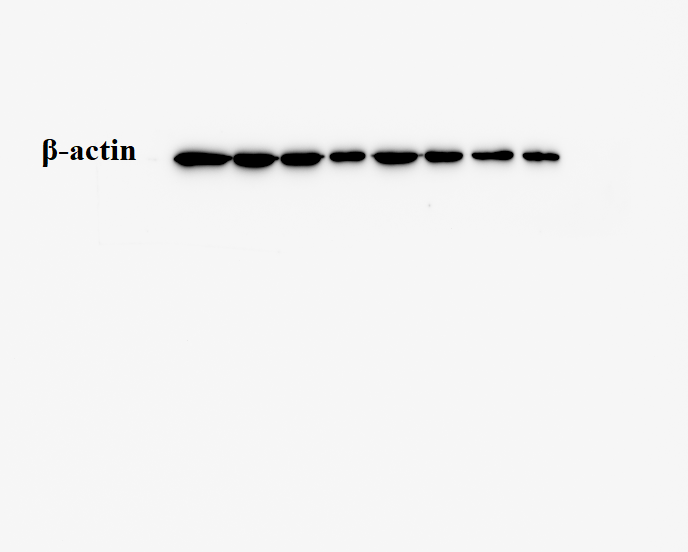

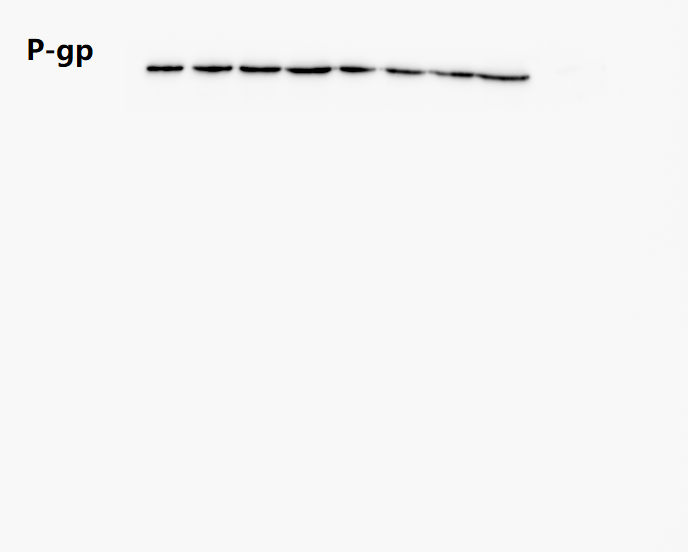


**The full-length blots of Fig. 18.**


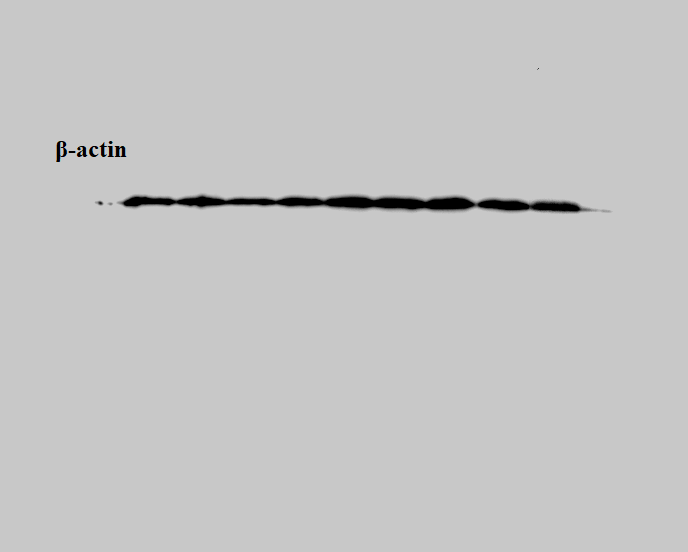

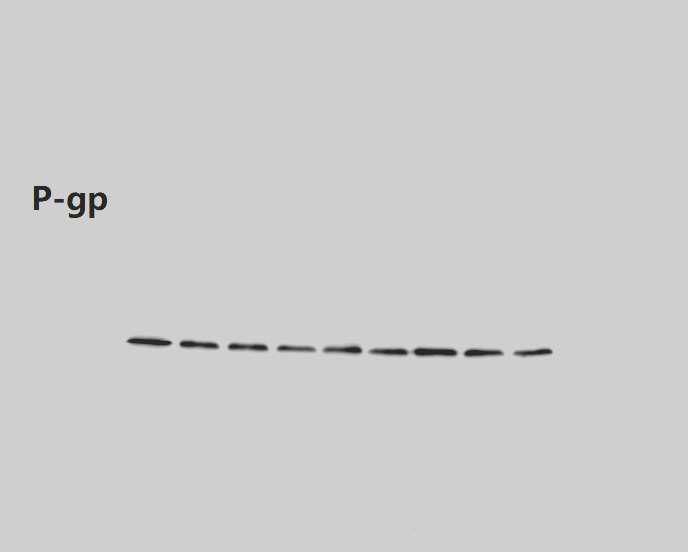


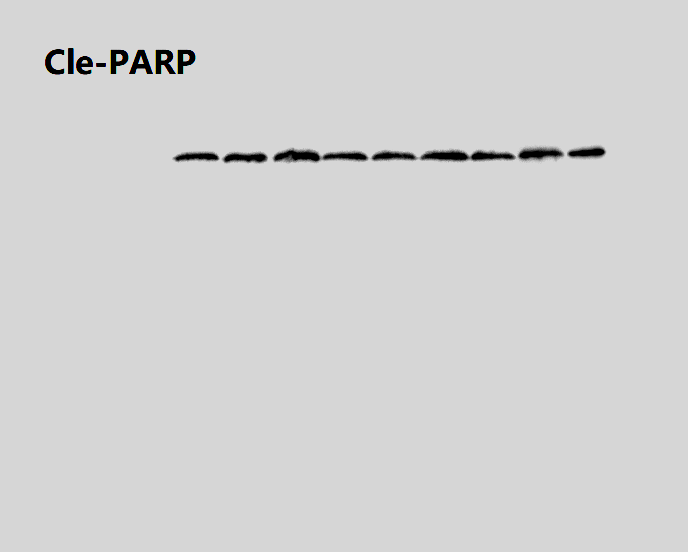

Supplement: Supplementary file 1 — Supplementary Information. [file 41598_2021_97676_MOESM1_ESM.doc]
